# Isorhamnetin ameliorates active ulcerative colitis potentially involving the Nrf2-PTGS2 axis: an integrative study from clinical evidence mining to in vivo validation

**DOI:** 10.1007/s00210-026-05737-4

**Published:** 2026-07-25

**Authors:** Zhenjia Fan, Luqing Zhao, Ning Ding, Yichong Wang, Danyan Li, Shengsheng Zhang

**Affiliations:** 1 Digestive Disease Center, Beijing Hospital of Traditional Chinese Medicine, Capital Medical University, Beijing, 100010 China; 2 Intensive Care Unit, Longhua Hospital, Shanghai University of Traditional Chinese Medicine, Shanghai, 200032 China

**Keywords:** Ulcerative colitis, TCM, Meta‐analysis, Isorhamnetin, Network pharmacology

## Abstract

Ulcerative colitis (UC) is a worldwide health issue with limited therapies. Traditional Chinese Medicine (TCM) shows potential, but lacks systematic efficacy evaluation and detailed mechanistic explanations. This study aims to evaluate the efficacy of TCM for active UC and explore its mechanisms. A meta-analysis of RCTs was conducted to assess TCM efficacy in active UC, and treatment efficacy was ranked. Core Chinese herbs were identified via association rule analysis. Network pharmacology and molecular docking predicted active components, targets, and pathways. Efficacy of the key component was validated in active UC mice. 17 studies (1,598 patients) showed TCM significantly improved active UC (SMD -1.73, 95%CI -2.21 to -1.25). Eight core Chinese herbs were identified. Network pharmacology revealed 76 overlapping targets. Isorhamnetin (Iso) showed strong binding to PTGS2 (binding energy: -9.34 kcal/mol) and Nrf2 (binding energy: -9.16 kcal/mol). In vivo, Iso dose-dependently alleviated disease symptoms, pathological damage, and spleen index in UC mice. TCM could ameliorate active UC. The key active component Iso shows therapeutic effects, with mechanisms potentially involving the Nrf2-PTGS2 axis and immune-inflammatory pathways. The findings offer some insight into the scientific basis of TCM in treating UC and predict that Iso may be a candidate for further mechanistic and clinical investigation.

**Supplementary information:**

The online version contains supplementary material available at https://doi.org/10.1007/s00210-026-05737-4.

## Introduction

Ulcerative colitis (UC) is a chronic and nonspecific inflammatory bowel disease (IBD) mainly involving the colorectal mucosa. It is typified by alternating episodes of remission and relapse, with symptoms including weight loss, diarrhea, and rectal bleeding (Shen et al. 2025). Currently, there are over 5 million UC patients worldwide. The prevalence in North America and Northern Europe has remained at a high level consistently. Moreover, the prevalence in Asia is showing a rapidly increasing trend and is projected to quadruple by 2035. This makes UC a global public health issue that needs to be addressed immediately (Le Berre et al. 2023).

UC pathogenesis is driven by the complex interplay of immune dysregulation, impaired intestinal mucosal barrier function, microbial dysbiosis, and genetic predisposition, ultimately leading to persistent inflammatory immune responses within the gut mucosa (Wangchuk et al. 2024; Long et al. 2024a, b). In terms of clinical treatment, the current medications are mainly 5-aminosalicylic acid preparations (5-ASA), corticosteroids, and immunosuppressants. However, these treatments have many limitations such as inducing metabolic disorders and increasing the risk of infection (Gros and Kaplan 2023; Singh et al. 2024; Nakase et al. 2022). Moreover, the clinical remission rate of patients is still less than 30% to 60% with standardized treatment, and some patients eventually experience treatment failure or disease progression requiring surgery (Shen et al. 2025; Lv et al. 2025). Therefore, within the current treatment framework, how to improve efficacy, reduce adverse effects, and delay disease progression is a core challenge in the management of UC.

Traditional Chinese Medicine (TCM) has long-standing experience in UC management. Characterized by the holistic concept and syndrome differentiation-based treatment, TCM employs various treatment methods such as “clearing heat and resolving dampness, strengthening the spleen and supplementing qi, activating blood circulation and resolving stasis”. It shows unique advantages in alleviating clinical symptoms, promoting mucosal healing, and reducing the relapse rate (Zhang et al. 2024). Numerous trials have reported the efficacy of single herbs and compound formulas for active UC (Yang et al. 2026; Zhang et al. 2026; Xu et al. 2026; Mo et al. 2022). However, several challenges remain. On the one hand, despite the abundance of clinical studies, there is a lack of systematic efficacy evaluations. On the other hand, TCM is characterized by the complexity of multiple components, multiple targets, and interactions across various pathways, but its key active components and mechanisms remain unclear. These limit the application and widespread adoption of TCM in modern medicine. Therefore, there is a requirement to systematically evaluate its clinical efficacy and deeply elucidate the key active components and molecular mechanisms of TCM for active UC, providing evidence for its clinical adoption.

**Fig. 1:**
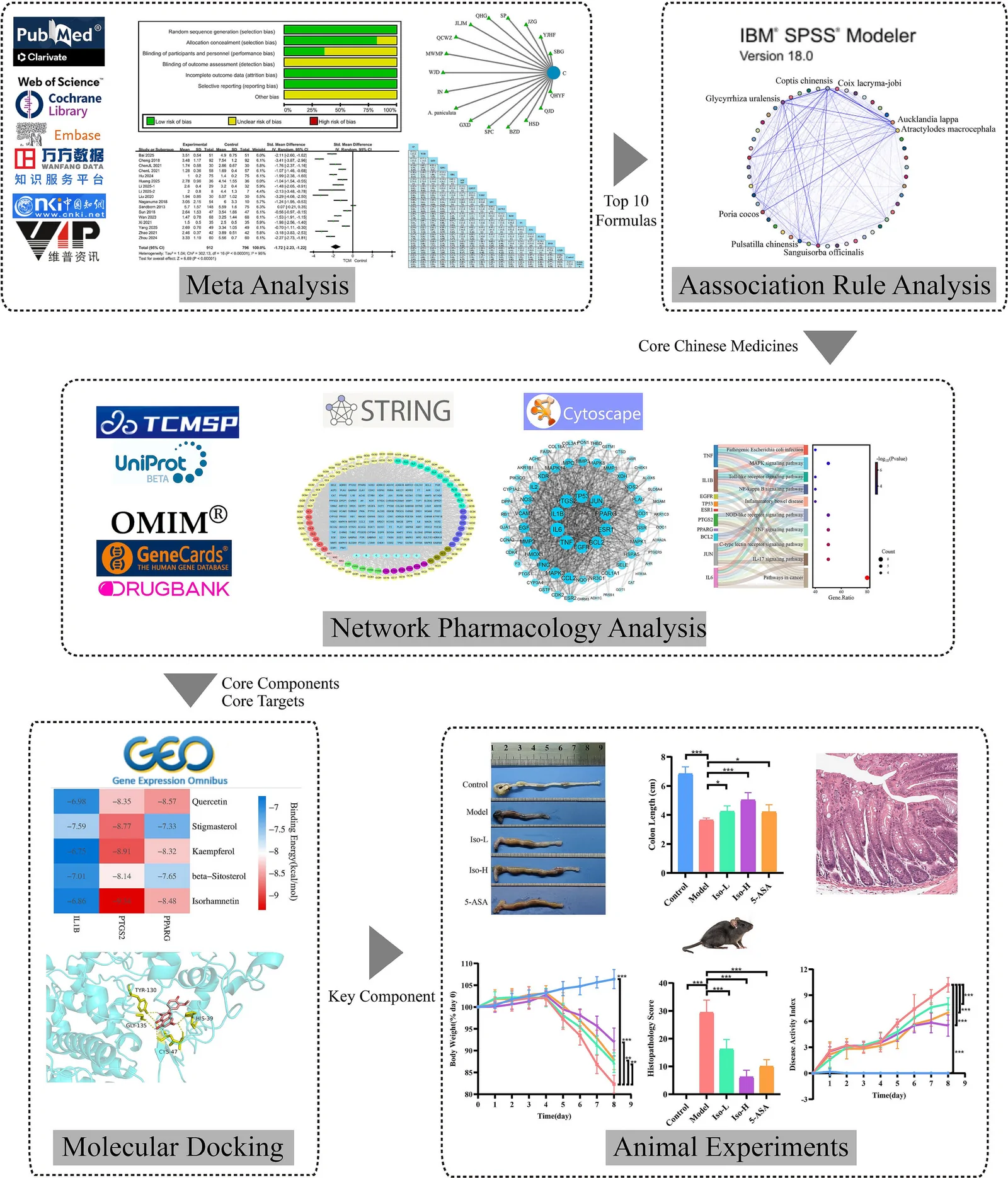
Flowchart of the study

We aim to systematically investigate the efficacy and mechanisms of TCM in treating active UC using a multi-method integrated research approach. First, we conduct a meta-analysis to evaluate the clinical efficacy of TCM for active UC in randomized controlled trials (RCTs), and the efficacy is ranked according to the Surface Under the Cumulative Ranking Curve (SUCRA). Second, we select the key bioactive component of TCM and predict key targets and pathways by association rule analysis, network pharmacology, and molecular docking techniques. Third, we test the effect of the key bioactive component through in vivo experiments. This study employs a comprehensive approach of “clinical evidence integration – active component screening – mechanism prediction – experimental validation”, to explore the scientific evidence of TCM for active UC, thereby providing new insights for drug discovery and precision therapy of UC. The detailed study process is depicted in Fig. 1.

## Materials and methods

### Meta-analysis

We registered the study protocol in the International Prospective Register of Systematic Reviews, PROSPERO (registration number: CRD 420251176591). The full protocol is available from https://www.crd.york.ac.uk/PROSPERO/view/CRD420251176591. The study was performed according to PRISMA guidelines (Page et al. 2021).

### Search strategy

A systematic study search was performed in four English databases: PubMed, Embase, CENTRAL, Web of Science; and three Chinese databases: CNKI, VIP, and *Wanfang*. We searched records from the creation of databases to June 2025. The searching keywords included “ulcerative colitis”, “inflammatory bowel disease”, “UC”, “IBD”, “Chinese medicine”, “TCM”, “traditional medicine”, “herb”, “decoction”, and “randomized controlled trial” (Supplementary Table 1).

### Eligibility criteria

The inclusion and exclusion criteria were formulated based on the PICOS framework:

P (Participants): Patients had a diagnosis of active UC (as diagnosed using any recognised diagnostic criteria) and were at least 16 years old. Disease severity at baseline was categorized using the Mayo score into three subgroups: Mild ulcerative colitis (MiUCs: Mayo score 3–5), Moderate ulcerative colitis (MoUCs: Mayo score 6–10), and Severe ulcerative colitis (SUCs: Mayo score 11–12). Patients with other intestinal diseases (such as infectious colitis, colorectal cancer) or inadequate hepatic and renal function were excluded.

I (Intervention): Oral TCM, administered in conjunction with conventional treatment. The composition of the TCM formula had to be explicitly described. For the purpose of network meta-analysis (NMA), TCM interventions were grouped into a node only if their herbal compositions were exactly identical. Therapies other than oral administration (such as enemas, acupuncture, injections) were excluded to maintain route-of-administration consistency.

C (Comparison): Conventional treatment (5-ASA, corticosteroids, etc.), with or without concomitant placebo.

O (Outcomes): Studies were included if they reported the Mayo score or the modified Mayo score, which are widely used to assess disease activity in UC.

S (Study Design): Randomized controlled trials (RCTs). The treatment course had to be ≥ 4 weeks to ensure an adequate observation period for efficacy.

Two reviewers independently screened and selected articles, and any discrepancies in the assessments were resolved with the third reviewer.

### Data extraction

Information was extracted as follows: first author, publication year, age, sample size, gender, disease course, severity grade, formula name, formula composition, treatment duration, and Mayo score. Two reviewers independently extracted data, and any discrepancies in the extraction were resolved with the third reviewer.

### Risk of bias

Two reviewers independently assessed the risk of bias using the Cochrane Collaboration Assessment Tool with the Review Manager 5.3. The following domains were evaluated for each included study: random sequence generation, allocation concealment, blinding of participants and personnel, blinding of outcome assessment, incomplete outcome data, selective reporting, and other biases. Each domain was judged as “low risk,” “unclear risk,” or “high risk.” Any discrepancies in the assessments were resolved with the third reviewer.

### Sensitivity analysis

To evaluate the stability of the primary findings, a leave-one-out sensitivity analysis was performed. Each study was successively removed from the NMA, and the pooled effect estimate was recalculated to see whether any single trial had an obvious influence on the overall results.

### Statistical analysis

A frequentist NMA was performed to synthesize the evidence from all included RCTs. The analysis was conducted using Stata 17.0, primarily employing the *network* suite of commands together with the *mvmeta* package for multivariate meta-analysis. All analyses employed the software's standard estimation commands. Since all comparisons used the same conventional treatment as the reference, the network was star-shaped with no closed loops. Consequently, a consistency model was specified, and no inconsistency assessment (e.g., node‑splitting, design‑by‑treatment interaction) was applicable. For the continuous variable Mayo score, the evaluation was done using the standardized mean difference (SMD) and 95% confidence intervals (CIs). Given the expected clinical heterogeneity among the included TCM formulas and patient populations, a random-effects model was adopted a priori. Heterogeneity was quantified using the *I*^2^ statistic. All included studies were two-arm trials; therefore, no special adjustments for multi-arm trial correlation structures were required. Potential publication bias was assessed using a funnel plot. Transitivity was evaluated by comparing the distribution of effect modifiers—disease severity, duration, and concomitant conventional therapy—across the included studies. Additionally, the SUCRA was estimated for each treatment node to rank the relative efficacy. Top ten ranked treatments provided a basis for subsequent research. Along with the SUCRA rankings, we reported the absolute effect of each treatment node compared to conventional treatment as SMD and 95% CIs. These estimates show the practical benefit of each treatment.

### Network node definition

In the NMA, each node represents a distinct TCM oral formula. To ensure clinical homogeneity and chemical composition consistency within each node, a rule was applied: different formulas were grouped into the same node only if their herbal compositions were exactly identical. No statistical clustering or merging of heterogeneous formulas was performed. The detailed mapping of each study's treatment arms to the assigned node labels, and herbal composition of each node are reported in Supplementary Table 2.

### Core Chinese herbs mining based on association rule analysis

Based on the results of SUCRA, the top ten ranked treatments were subjected to association rule analysis to find core Chinese herbs potentially effective for active UC. Firstly, the Chinese medicines contained within the top ten ranked treatments were unified following the *Pharmacopoeia of the People’s Republic*. Secondly, association rule analysis was completed using IBM SPSS Modeler 18.0 to generate the Chinese medicines correlation network and to calculate the support and confidence percentages for each frequent itemset. Finally, the core Chinese herbs were determined based on thresholds of ≥ 50% for support percentages and ≥ 80% for confidence percentages.

### Network pharmacology analysis of the core Chinese herbs

#### Identification of active components and potential targets

Chemical components of core Chinese herbs were searched via the TCMSP Database, and active components were screened based on oral bioavailability (OB) ≥ 30% and drug likeness (DL) ≥ 0.18. The gene targets of active components were annotated using the “Homo sapiens” species filter in the Uniprot database. UC-related human target genes were identified from the GeneCards, OMIM, and DrugBank databases. Overlapping targets of UC and active components were obtained via Venn diagram. The core Chinese herbs, active components, and overlapping targets were inputted into Cytoscape v3.8.2 to construct a “Chinese herb – component – target” network related to UC.

#### Construction of protein–protein interaction (PPI) network

Overlapping targets were submitted into STRING database for PPI network construction with *Homo sapiens* and *medium confidence (0.400)*. We performed a topological analysis of the PPI network via the cytoHubba plugin in Cytoscape v3.8.2 and visualized the results. An integrated analysis using Degree, Maximum Neighborhood Component (MNC), and Maximal Clique Centrality (MCC) was performed to select the top ten core targets.

#### GO and KEGG analysis

Enrichment analysis of the ten core targets was conducted via the DAVID database, with a data selection threshold of *p* < 0.05, and the top ten records were visualized to show the molecular features of TCM for UC.

### Molecular docking

We downloaded datasets of active-phase UC (Series: GSE87466 and GSE206285, Platform: GPL13158) via the GEO database, and obtained differentially expressed genes (DEGs) between human normal and UC colonic tissue based on |fold change|≥ 1.5 and *p* < 0.05. The intersection was taken between these DEGs and the ten core targets acquired in Sect. 2.3.2 to obtain the key targets. We searched for the key target protein receptor’s 3D structures from the PDB database.

We performed a topological analysis of the “Chinese herb – component – target” network in 2.3.1 using the cytoHubba plugin within Cytoscape v3.8.2, and screened the top five core components based on their “Degree” score. The chemical structures of ligands were downloaded via the PubChem database.

We carried out molecular docking of protein receptors and ligands via AutoDock Vina v1.2.5 to obtain the binding energy, and visualized the pair with the strongest binding energy using PyMOL.

Additionally, we predicted the binding sites of Nrf2 within the PTGS2 promoter using the JASPAR database.

### Animal studies

#### Reagents

Isorhamnetin (Iso) (> 98% purity) was obtained from Macklin Biochemical Technology Co., Ltd. Mesalazine, a type of 5-ASA, was obtained from Sunflower Pharmaceutical Group Co., Ltd. Dextran Sulfate Sodium (DSS, batch number: 160110, molecular weight: 36,000—50,000 Da) was obtained from MP Biomedicals (Irvine, CA, USA).

#### Experimental animals and study design

Thirty male C57BL/6 mice (8 weeks, 20–22 g) were obtained from SPF (Beijing) Biotechnology Co., Ltd. All animal procedures were approved by the Animal Ethics Committee of the Beijing Institute of Chinese Medicine (No. BJTCM-M-2025–11-05). This study is reported in compliance with the Animal Research Reporting of In Vivo Experiments (ARRIVE) guidelines.

Mice were reared in standard conditions (pathogen-free, 23–25 °C, 50–70% humidity, a 12-h light/dark cycle) with ad libitum food and water. Mice were randomly allocated into five groups using a computer-generated random number table: the normal control (Control) group, the model (Model) group, the low-dose isorhamnetin (Iso-L) group, the high-dose isorhamnetin (Iso-H) group, and the 5-ASA (5-ASA) group (*n *= 6 each). The sample size of *n *= 6 per group was determined based on previous studies (Wang et al. 2024).

Active UC was induced by administering 3% DSS (w/v) solution, prepared in sterilized drinking water, freely available for 7 consecutive days since Day 0 to induce UC. The Control group received normal sterilized drinking water. Then, the DSS solution was replaced with normal sterilized drinking water.

Isorhamnetin was formulated as a suspension in 0.5% carboxymethylcellulose sodium (CMC-Na) (Kong et al. 2025). Starting from Day 0, the Iso-L and Iso-H groups were treated with Iso at doses of 25 mg/kg and 50 mg/kg intragastrically once daily for 7 days. 5-ASA was similarly prepared and administered at 100 mg/kg. The Control and Model groups received an equal volume of 0.5% CMC-Na solution. Each mouse was given the solution intragastrically at 0.1 mL/10 g. The dosages of Iso and 5-ASA were referenced from previously published literature showing anti-inflammatory efficacy in murine models (Wang et al. 2024; Ren et al. 2023).

On Day 8, mice were anesthetized by inhalation of isoflurane and subsequently euthanized through cervical dislocation. We collected colon tissue and measured its length. The researchers performing the clinical scoring and statistical analyses were blinded to the group allocation to reduce the risk of bias.

The primary endpoints were: (1) body weight change, (2) Disease Activity Index (DAI) score, (3) colon length, (4) colonic histopathological score, and (5) spleen index.

#### Weight and DAI

Body weight was recorded and fecal samples were collected for the assessment of the DAI to evaluate disease severity at the same time each day prior to gavage. The DAI is a composite score as shown in Supplementary Table 3 (Wu et al. 2025).

#### H&E staining and histopathological assessment

Tissues from the distal one-third of colon were fixed, paraffin-embedded, sectioned (5 μm), and H&E-stained. Stained sections were examined microscopically for histopathological assessment. The criteria of histological scores for colitis are shown in Supplementary Table 4 (Wu et al. 2025).

#### Statistical analysis

Data were presented as mean ± standard deviation (SD) and analyzed with SPSS 26.0. Graphs were made using GraphPad Prism 9.5.1. The primary analysis was pre-specified to compare each treatment group with the Model group across all endpoints. Comparisons between multiple groups were performed using one-way analysis of variance (ANOVA). When ANOVA yielded a significant result, post hoc pairwise comparisons were conducted using the least significant difference (LSD) test. A two-tailed alpha level of 0.05 was used for all analyses. Statistical significance was defined as *p* < 0.05. Effect sizes for outcomes were reported as mean differences with 95% CIs.

## Outcomes

### Search results

**Fig. 2:**
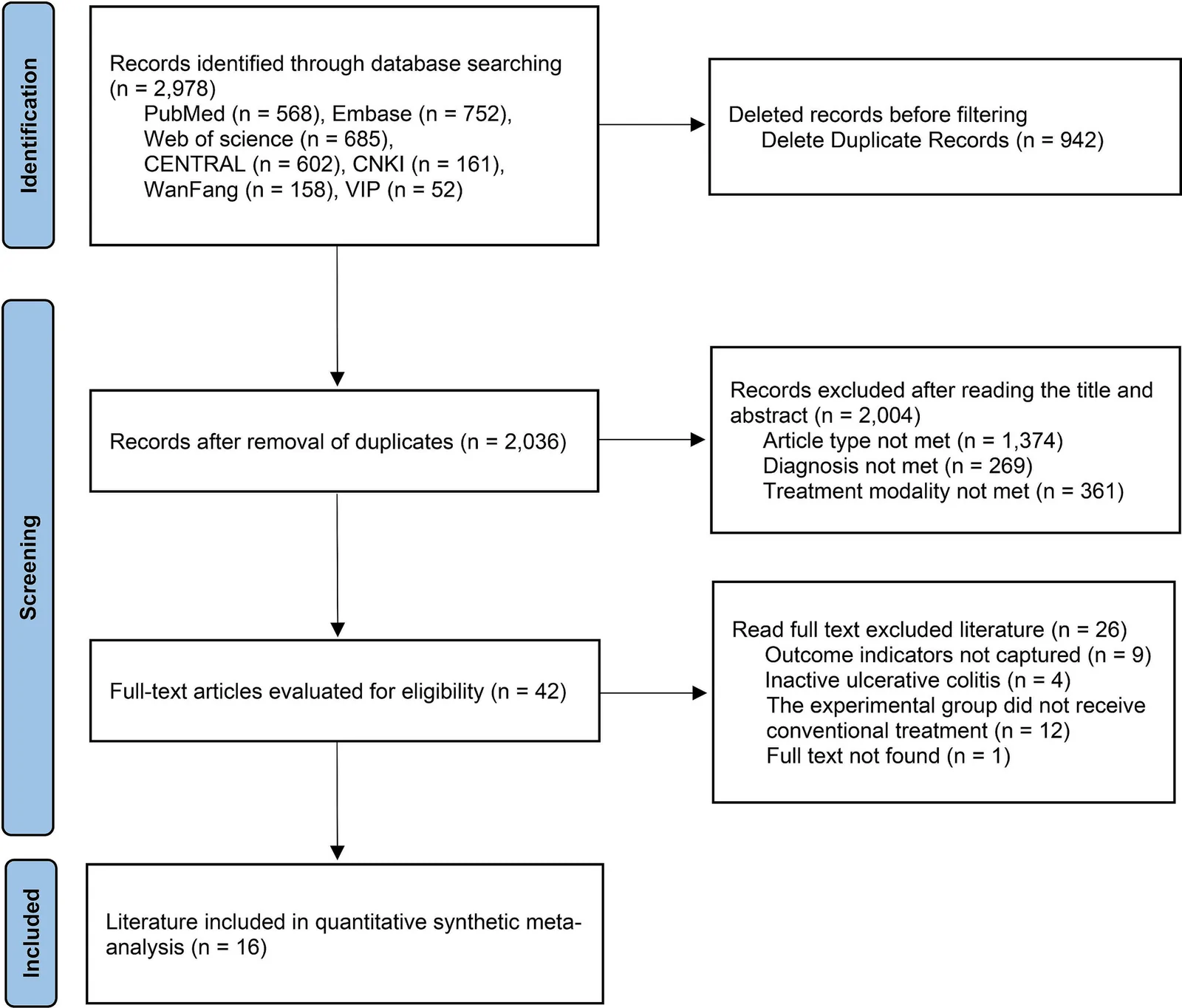
PRISMA flow diagram

According to the predefined search strategy, 2,978 publications were identified. After duplicate removal (*n *= 942) and title/abstract screening (*n *= 2,004), 42 articles underwent full-text review. Finally, 16 articles meeting the inclusion and exclusion criteria (Bai et al. 2025; Cheng et al. 2018; Chen et al. 2021a, b; Hu et al. 2024; Huang et al. 2025; Li et al. 2025; Liu et al. 2020; Naganuma et al. 2018; Sandborn et al. 2013; Sun and Zhang 2018; Wan et al. 2023; Xi et al. 2021; Yang and Hu 2025; Zhao et al. 2021; Zhou et al. 2024), one of which contained two independent studies (Li et al. 2025). Therefore, 17 studies were included in the NMA. The screening process is detailed in the PRISMA flow diagram (Fig. 2).

### Study characteristics and risk of bias

Network diagram is presented in Supplementary Fig. 1. A total of 1,598 patients from the 17 included studies were analyzed, with 796 in the control group and 802 in the trial group. The characteristics of the trial and control groups were comparable. The treatment duration in all studies ranged from 28 to 168 days. In terms of UC severity, MiUCs, MoUCs, and SUCs were included. The details are shown in Supplementary Table 5.

**Fig. 3:**
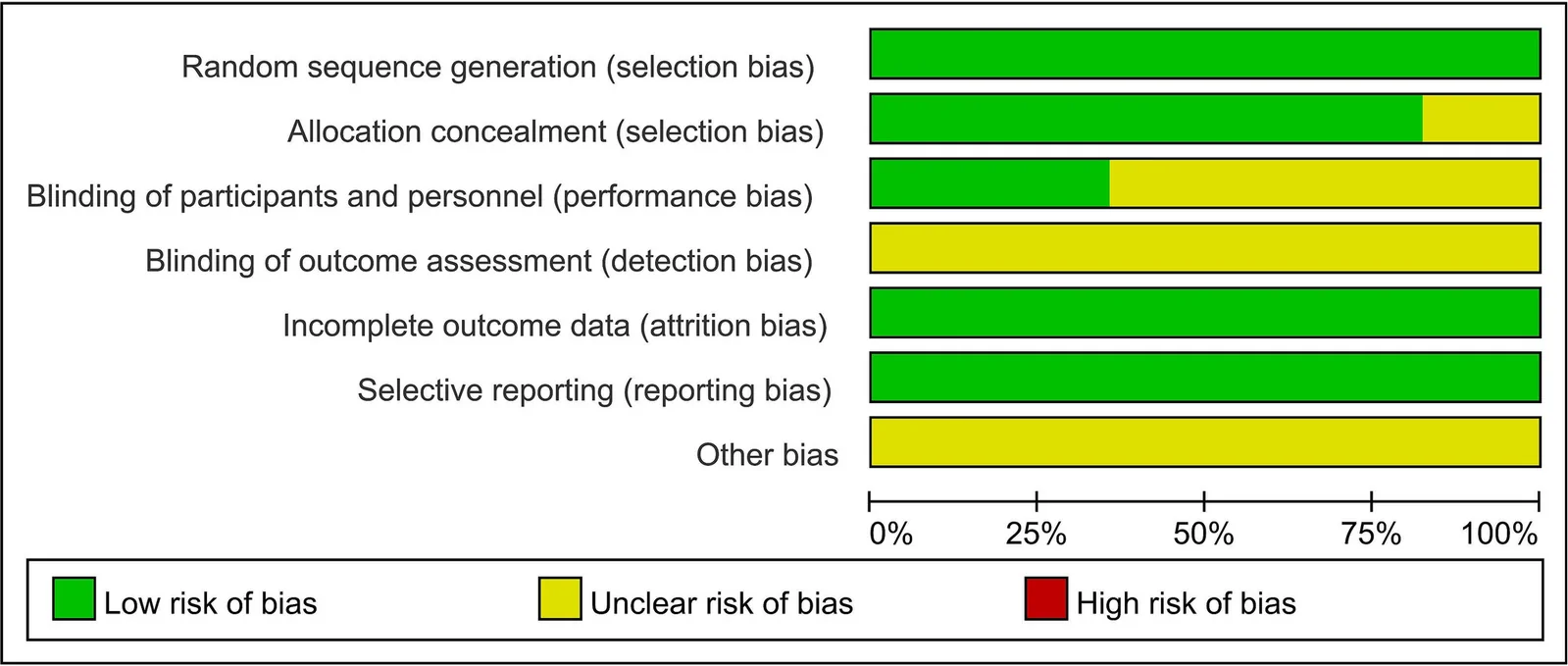
Risk of bias graph

All studies employed random allocation. Among these, 14 studies employed random number tables or computer-generated randomization, while 3 studies (Cheng et al. 2018; Liu et al. 2020; Sun and Zhang 2018) did not report the details of randomization. 6 studies employed double-blinded trials (Huang et al. 2025; Li et al. 2025; Naganuma et al. 2018; Sandborn et al. 2013; Zhou et al. 2024). Complete data and reliable results were available for all included studies. Overall, all studies were assessed as either “low risk” or “unclear risk” across the key domains of random sequence generation, allocation concealment, and blinding. Notably, no study was judged at “high risk” of bias in these domains, suggesting an acceptable baseline quality for evidence synthesis. However, the “unclear risk” rating for allocation concealment and blinding may lead to an overestimation of the treatment effect. Given this uncertainty, we present the SUCRA rankings as suggesting a class of potentially comparable formulas, rather than as evidence of superiority for any single one. A figure of the risk of bias is presented in Fig. 3, and the detailed judgments are provided in Supplementary Fig. 2.

### Meta-analysis results

**Fig. 4:**
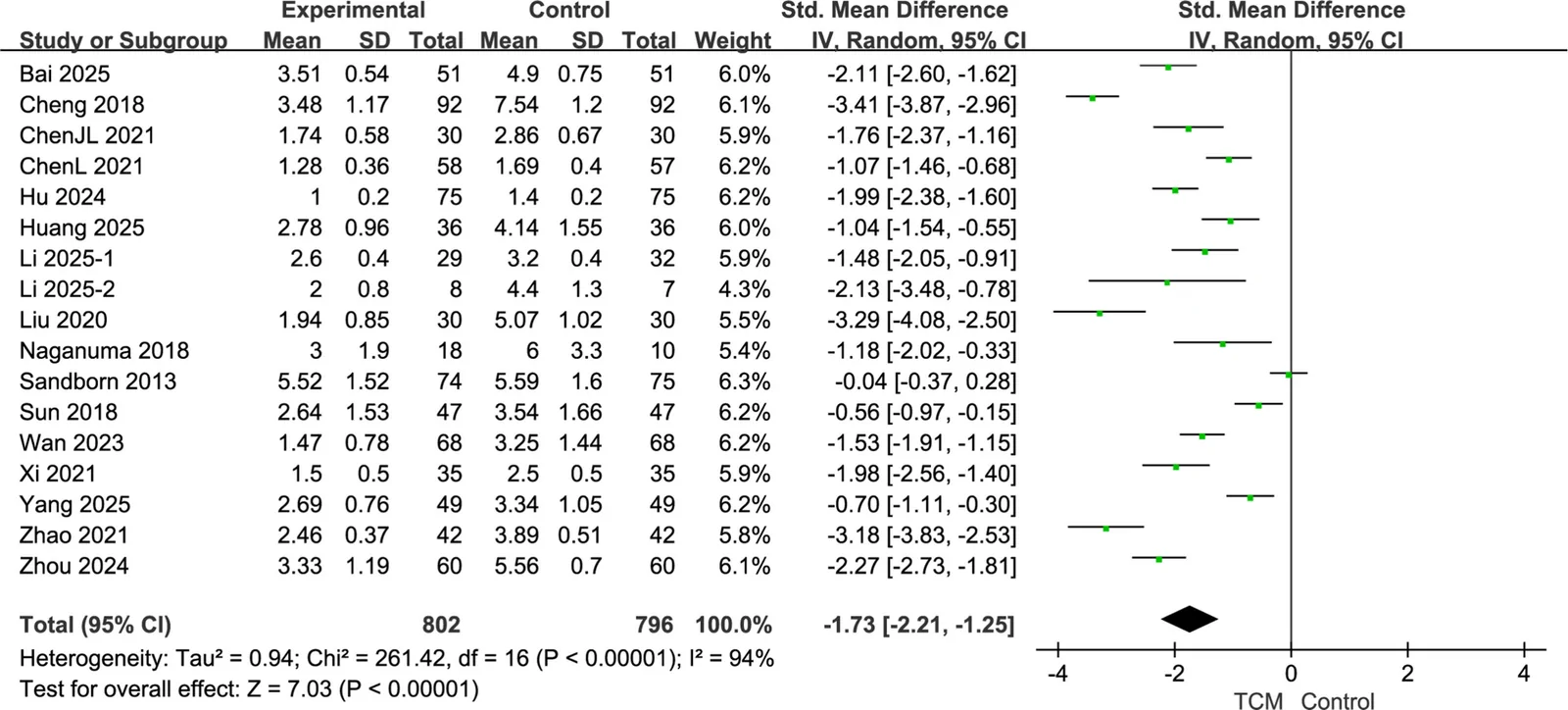
Forest plot of Mayo score

The analysis results (Fig. 4) showed that patients treated with TCM had a significantly lower Mayo score than those receiving conventional treatment alone (SMD −1.73, 95%CI −2.21 to −1.25). Significant heterogeneity was observed (*p* < 0.00001, *I*^2^ = 94%). The funnel plot indicated potential asymmetry, suggesting possible publication bias (Supplementary Fig. 3). Given the limited number of included studies, results should be interpreted cautiously.

**Fig. 5:**
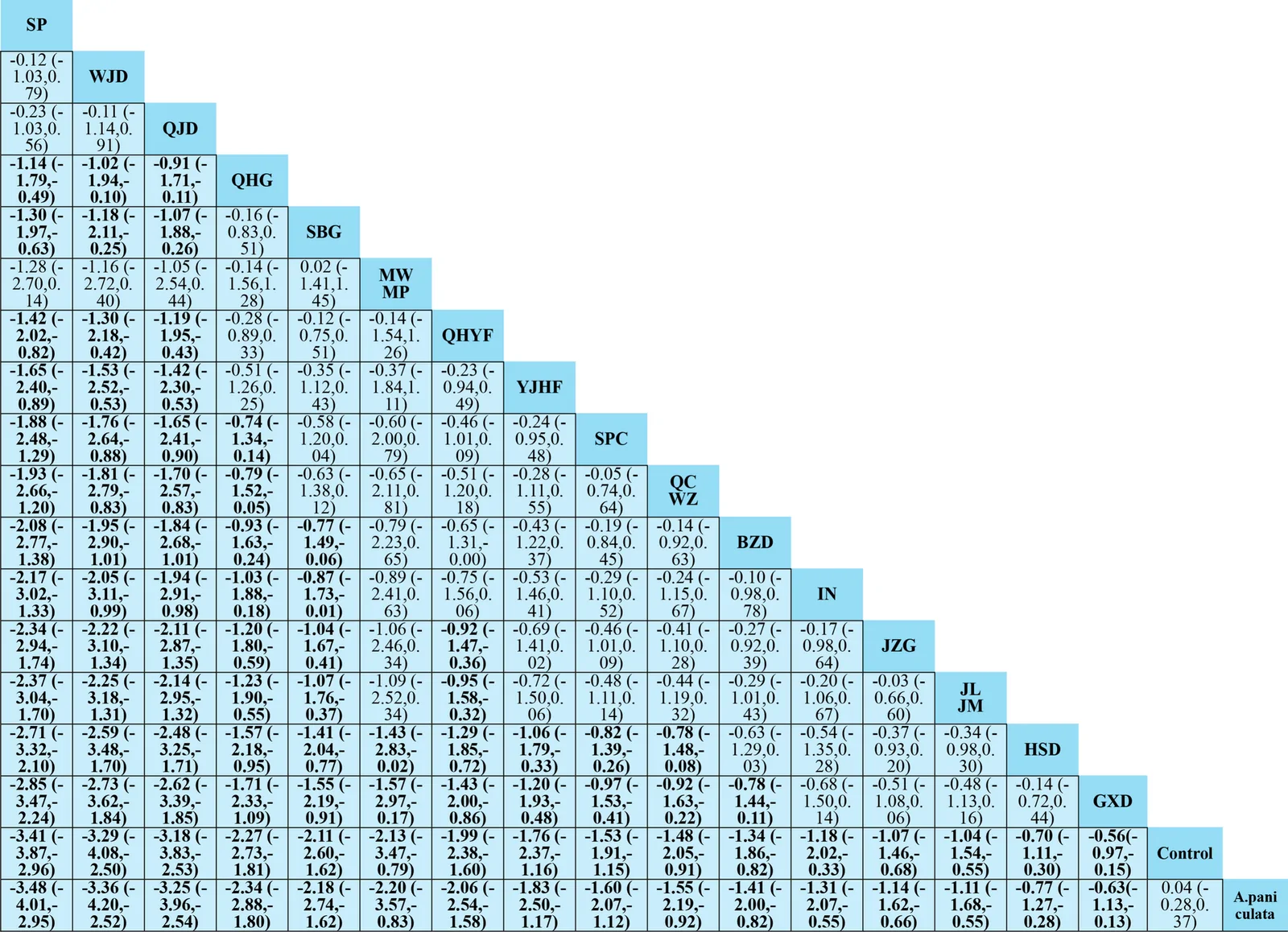
Comprehensive estimate for the comparison of outcomes. A. paniculata: Andrographis paniculata (Chuanxinlian, Sandborn 2013), BZD: Buyang Zhixie Decoction (Xi et al. 2021), C: conventional treatment, GXD: Gancao Xiexin Decoction (Sun and Zhang 2018), HSD: Huangqin Shaoyao Decoction (Yang and Hu 2025), IN: Indigo Naturalis (Qingdai, Naganuma 2018), JLJM: Jianpi Lishi Jiedu Mixture (Huang et al. 2025), JZG: Jianpi Zhixiening Granule (Chen 2021b), MWMP: Wumei Pill (Li –2), QCWZ: Qingchang Wenzhong Decoction (Li 2025–1), QHG: Qingre Huashi Granule (Zhou et al. 2024), QHYF: Qingre Huashi Yuyang Formula (Hu et al. 2024), QJD: QingreJianpi Decoction (Zhao et al. 2021), SBG: Shenling Baizhu Granule (Bai et al. 2025), SP: Sishen Pill (Cheng et al. 2018), SPC: Sishen Pill Compound (Wan et al. 2023), WJD: Wenglian Jiedu Decoction (Liu et al. 2020), YJHF: Yiqi Jiedu Huayu Formula (Chen 2021a)

We examined the absolute effect estimates for the top-ranked TCM interventions versus conventional treatment. The pairwise effect estimates (SMD with 95% CI) for all treatment nodes are presented in Fig. 5. The SMDs of ten top-ranked TCM formulations ranged from −1.48 (QCWZ) to −3.41 (SP), with all 95% CIs excluding zero, suggesting statistically significant benefits for all ten formulations.

We further ranked the effectiveness of treatments using the SUCRA. The results showed that, for the Mayo score, the top ten formulations are SP (SUCRA = 95.8%), WJD (SUCRA = 93.2%), QJD (SUCRA = 91.8%), QHG **(**SUCRA = 76.4%), SBG (SUCRA = 71.5%), MWMP (SUCRA = 67.9%), QHYF (SUCRA = 67.7%), YJHF (SUCRA = 58.6%), SPC (SUCRA = 49.9%), and QCWZ (SUCRA = 47.9%) (Supplementary Fig. 4, Supplementary Table 6). Although SP, WJD, and QJD exhibited the highest SUCRA values, their 95% CIs showed considerable overlap (e.g., SP: −3.41 [95% CI −3.87 to −2.96]; WJD: −3.29 [−4.08 to −2.50]; QJD: −3.18 [−3.83 to −2.53]), suggesting that the differences among these top-ranked formulations are imprecise. These rankings should therefore not be interpreted as evidence of superiority; rather, they suggest a class of TCM formulations with comparable, potentially clinically meaningful efficacy relative to conventional treatment.

### Inconsistency assessment and sensitivity analysis

Because the network exhibited a star-shaped structure with all TCM nodes connected only through the common comparator (conventional treatment) and contained no closed loops, inconsistency between direct and indirect evidence could not be assessed. Node-splitting and global inconsistency tests were therefore not applicable.

The leave-one-out sensitivity analysis showed that the pooled effect estimate remained stable and statistically significant regardless of which individual study was removed from the network. This indicates that the findings are stable and not driven by any study.

### Transitivity assessment

The included studies were clinically comparable across key effect modifiers. Most trials enrolled patients with mild‑to‑moderate active UC (Mayo score range 3–10). Concomitant conventional therapy consisted mainly of oral 5‑ASA; a few studies permitted corticosteroids. The treatment duration of most trials ranged from 28 to 90 days. We found no evidence of systematic differences in these characteristics between studies. This supports the assumption of transitivity underlying the NMA. Detailed characteristics are provided in Supplementary Table 5.

### Core Chinese herbs mining

**Fig. 6:**
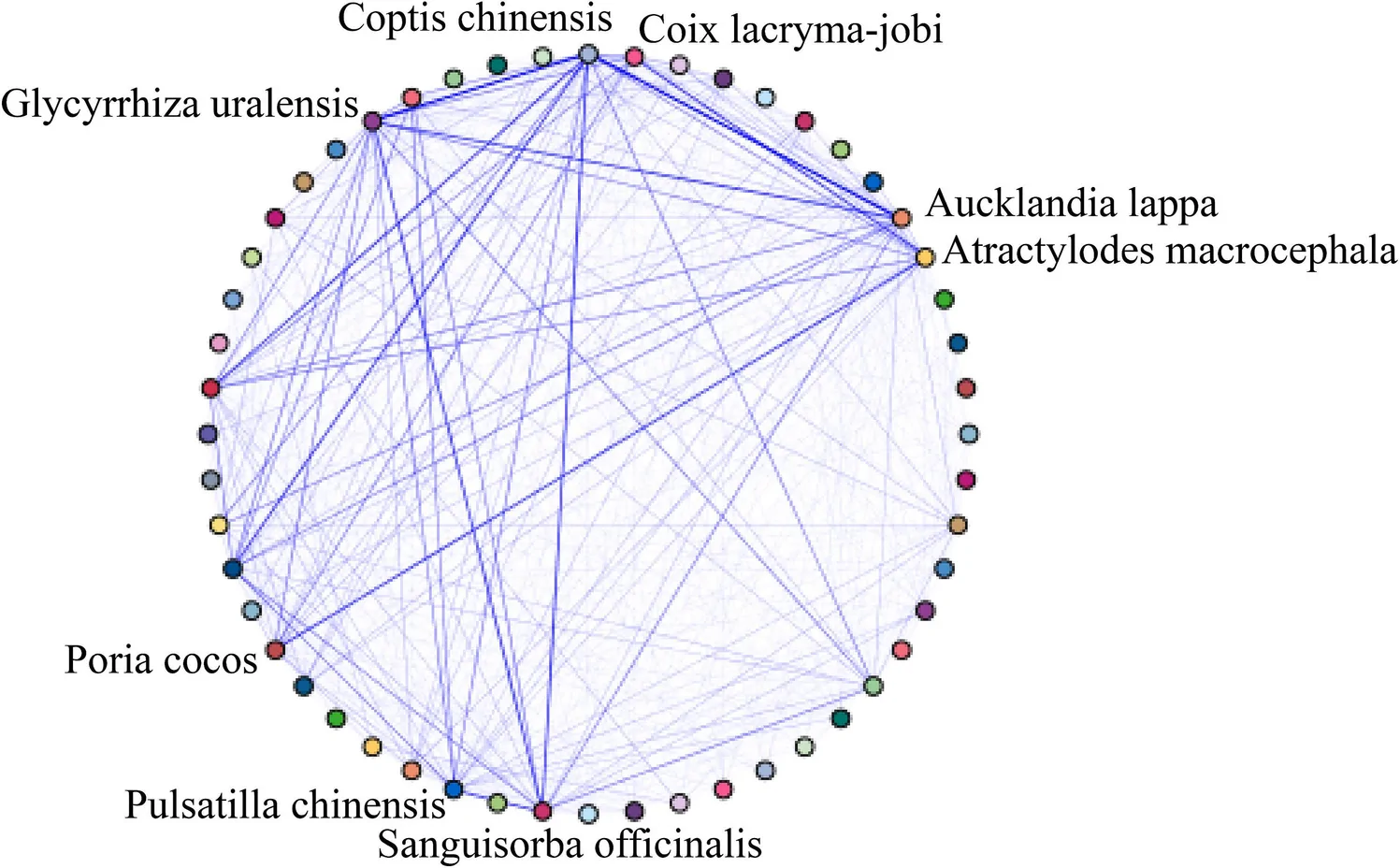
Network diagram of association rule analysis

The TCM formulations from the top ten treatments of the SUCRA ranking were collected, and their names were standardized, involving a total of 55 different herbs. We performed an association rule analysis on these herbs, with the network diagram presented in Fig. 6. With criteria set at ≥ 50% for support and ≥ 80% for confidence, eight herbs were identified as the core Chinese herbs: *Aucklandia lappa* (Mu Xiang), *Coptis chinensis* (Huang Lian), *Glycyrrhiza uralensis* (Gan Cao), *Coix lacryma-jobi* (Yi Yi Ren), *Poria cocos* (Fu Ling), *Pulsatilla chinensis* (Bai Tou Weng), *Sanguisorba officinalis* (Di Yu), and *Atractylodes macrocephala* (Bai Zhu) (Table 1).

**Table 1:** Association Rule Analysis of Chinese Medicine

Previous items	Back items	Support count	Support (%)	Confidence (%)
*Aucklandia lappa*	*Coptis chinensis*	6	60	83.33
*Coptis chinensis*	*Glycyrrhiza uralensis*	6	60	83.33
*Glycyrrhiza uralensis*	*Coptis chinensis*	6	60	83.33
*Coix lacryma-jobi*	*Atractylodes macrocephala*	5	50	80
*Poria cocos*	*Atractylodes macrocephala*	5	50	80
*Pulsatilla chinensis*	*Sanguisorba officinalis*	5	50	80
*Sanguisorba officinalis*	*Pulsatilla chinensis*	5	50	80
*Glycyrrhiza uralensis*	*Sanguisorba officinalis*	5	50	80
*Coptis chinensis*	*Sanguisorba officinalis*	5	50	80
*Glycyrrhiza uralensis*	*Aucklandia lappa*	5	50	80
*Coptis chinensis*	*Aucklandia lappa*	5	50	100
*Sanguisorba officinalis*	*Glycyrrhiza uralensis and Coptis chinensis*	5	50	80
*Glycyrrhiza uralensis*	*Aucklandia lappa and Coptis chinensis*	5	50	80
*Aucklandia lappa*	*Glycyrrhiza uralensis and Coptis chinensis*	5	50	80

### Targets analysis of core Chinese herbs and ulcerative colitis

**Fig. 7:**
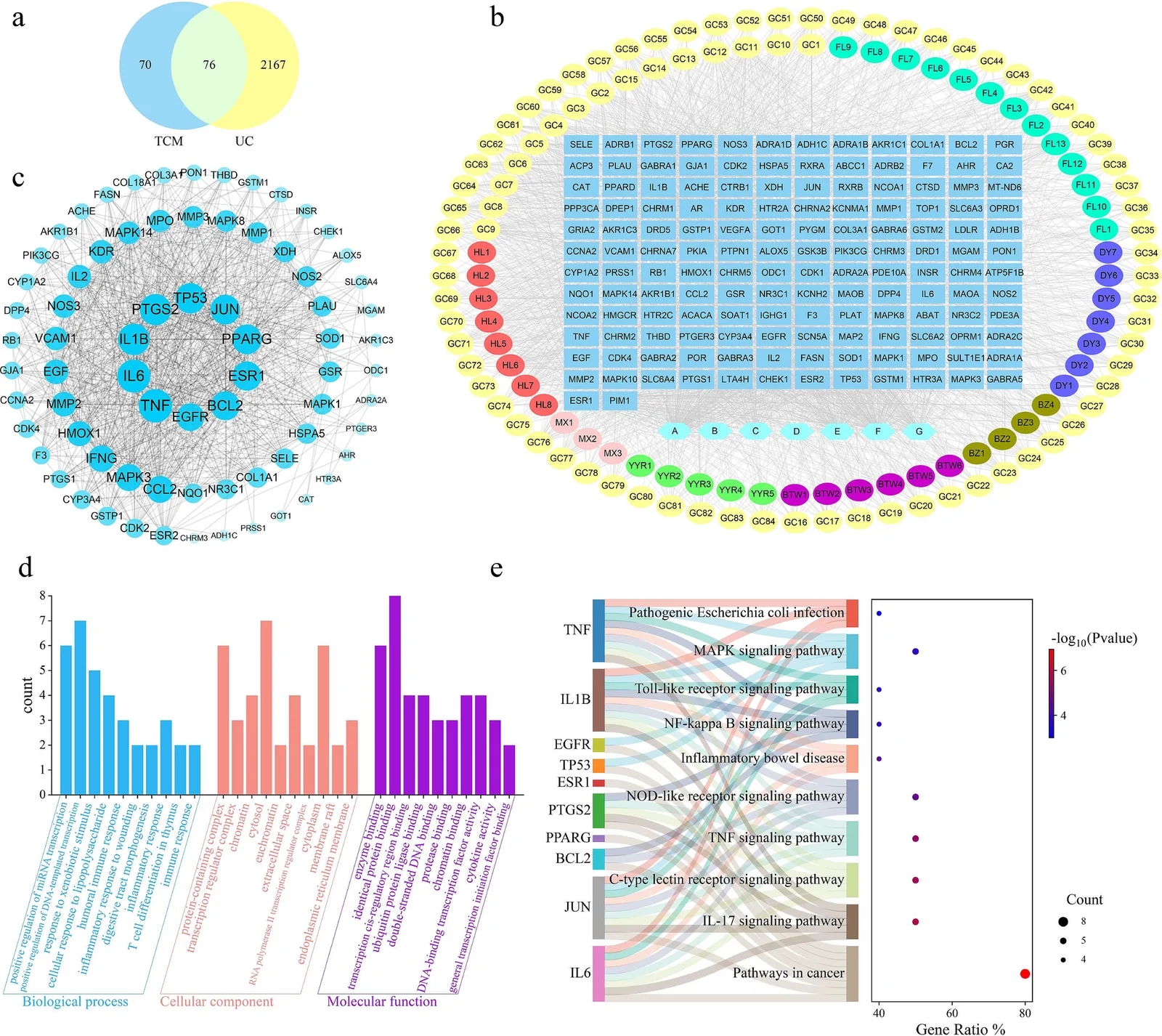
Results of the network pharmacology analysis on core Chinese herbs and UC. (a) Venn diagram of the core Chinese herbs and UC. (b) Network of Chinese herb – component – target. (c) PPI network. (d) GO analysis. (e) KEGG analysis. A-G: Active components present in two or more core Chinese herbs. A: Quercetin, B: Mairin, C: Isorhamnetin, D: Sitosterol, E: Kaempferol, F: Stigmasterol, G: beta-Sitosterol. MX: *Aucklandia lappa* (Mu Xiang), HL: *Coptis chinensis* (Huang Lian), GC: *Glycyrrhiza uralensis* (Gan Cao), YYR: *Coix lacryma-jobi* (Yi Yi Ren), FL: *Poria cocos* (Fu Ling), BTW: *Pulsatilla chinensis* (Bai Tou Weng), DY: *Sanguisorba officinalis* (Di Yu), BZ: *Atractylodes macrocephala* (Bai Zhu)

163 active components of the eight core Chinese herbs were identified in the TCMSP database: 6 from *Aucklandia lappa*, 10 from *Coptis chinensis*, 92 from *Glycyrrhiza uralensis*, 9 from *Coix lacryma-jobi*, 15 from *Poria cocos*, 11 from *Pulsatilla chinensis*, 13 from *Sanguisorba officinalis*, and 7 from *Atractylodes macrocephala*. Gene targets of active components were annotated using the UniProt database. After deduplication, 146 targets from active components of core Chinese herbs were obtained. 2,243 human target genes for UC were identified from GeneCards, OMIM, and DrugBank databases. The Venn diagram shows that the core Chinese herbs for UC have 76 overlapping targets (Fig. 7a). A “Chinese herb – component – target” network was generated with Cytoscape v3.8.2 (Fig. 7b).

### PPI network analysis

76 targets were analyzed using STRING (medium confidence, 0.400). After hiding two disconnected nodes, the PPI network was loaded into Cytoscape v3.8.2 for cytoHubba analysis, generating 74 nodes and 844 edges. The results are visualized in Fig. 7c. Based on an integrated analysis of Degree, MCC, and MNC values, ten core targets were selected: TNF, IL6, IL1B, PTGS2, TP53, JUN, PPARG, ESR1, BCL2, and EGFR.

### GO and KEGG analysis

The GO analysis results (Fig. 7d) indicated that Iso may exert anti-UC effects by regulating immune response, inflammatory response, digestive tract morphogenesis, T cell differentiation, chromatin, and extracellular space. KEGG analysis results (Fig. 7e) indicated that the IL-17, NF-κB, and MAPK signaling pathway may play important roles in UC.

### Molecular docking results

**Fig. 8:**
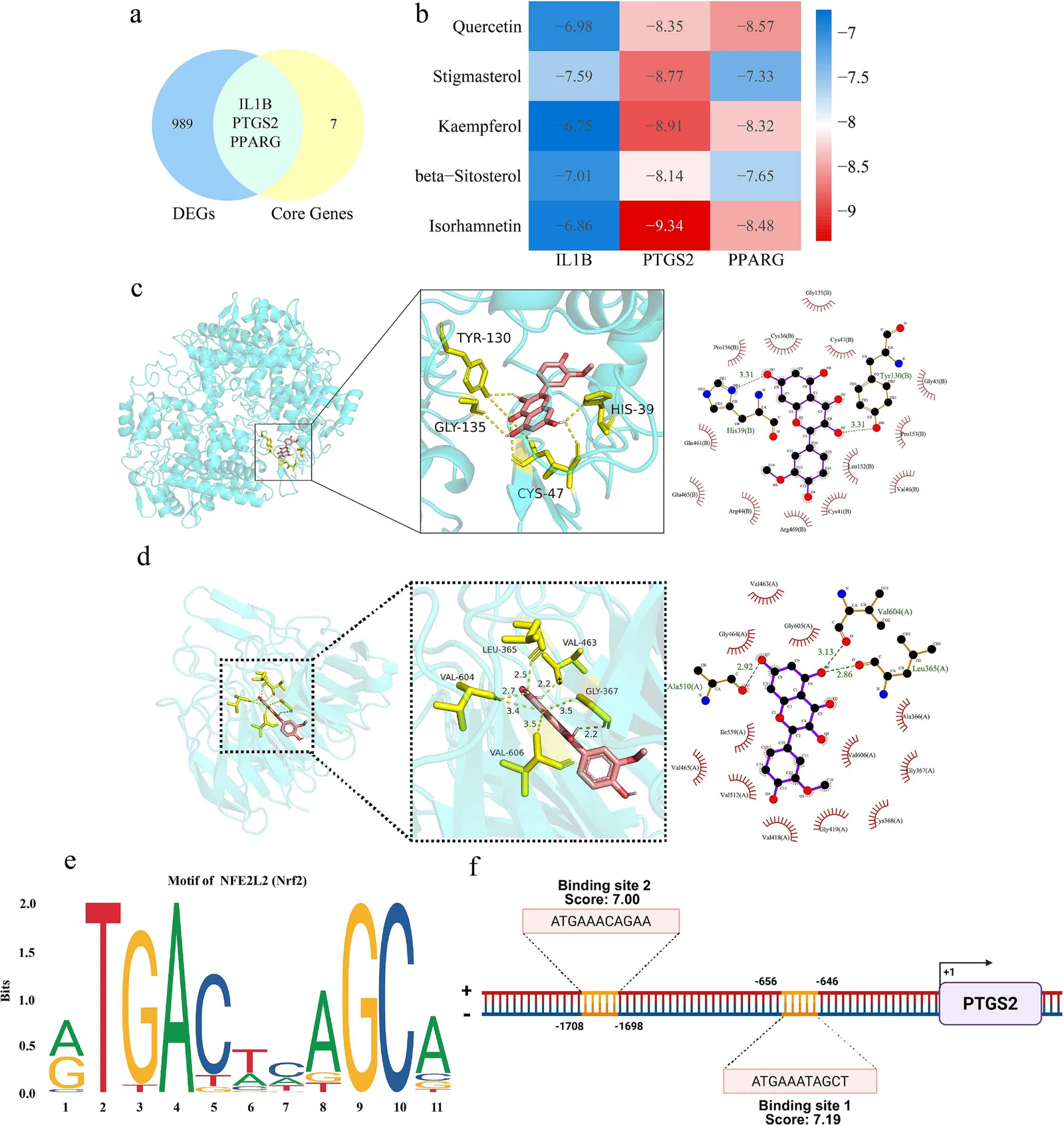
Results of molecular docking. (**a**) Venn analysis result between DEGs and core genes in the PPI network. (**b**) Heatmap of binding energies for active components and key targets. (**c**) Interaction of isorhamnetin with PTGS2. (**d**) Interaction of isorhamnetin with Nrf2. (**e**) The binding motif of Nrf2. (f) predict the binding sites between Nrf2 and PTGS2

A total of 992 DEGs between UC tissues and normal colonic tissues were obtained via the GEO datasets. The overlap between 992 DEGs and 10 core target genes from the PPI network yielded three potential key targets: IL1B, PTGS2, and PPARG (Fig. 8a). The 3D protein structures for these three key targets were obtained via the PDB database with the PDB IDs: 5R7W (IL1B), 5F19 (PTGS2), and 8ATY (PPARG). A cytoHubba analysis was performed on the “Chinese herb – component – target” network in Sect. 3.6. After sorting by decreasing “Degree” values, the top five core active components were: quercetin, stigmasterol, kaempferol, beta-sitosterol, and isorhamnetin (Iso).

Molecular docking was performed between the protein receptors of the three key targets and the top five core active components, with the binding energies shown in Fig. 8b. Iso exhibited the strongest binding energy with PTGS2 (binding energy: −9.34 kcal/mol). The binding interaction, depicted in Fig. 8c, shows that Iso forms hydrogen bonds with PTGS2 at TRY-130, HIS-39, GLY-135, and CYS-47.

Two previous studies have reported that Iso alleviates UC through the regulation of Nrf2 (Ru et al. 2024; Zhang et al. 2025). In addition, it has been reported that activation of Nrf2 can downregulate PTGS2 levels (Deng et al. 2025). Based on these findings, we try to explore the relationship between Nrf2 and PTGS2 in UC. Molecular docking results indicated that Iso binds to Nrf2 (PDB ID: 1X2R; binding energy: −9.16 kcal/mol) at LEU-365, VAL-463, VAL-604, VAL-606, and GLY-367, suggesting that Iso may activate Nrf2, which is consistent with previous studies (Fig. 8d). We further predict the interaction between Nrf2 and PTGS2 via the JASPAR database. The binding motif of Nrf2 was obtained (Fig. 8e), and potential Nrf2 binding sites within the PTGS2 promoter were then predicted using JASPAR. The results showed that there are two potential Nrf2 binding sites in the PTGS2 promoter region, suggesting that Nrf2 may regulate PTGS2 transcription (Fig. 8f).

Collectively, these findings raise the hypothesis that the Nrf2-PTGS2 axis may be involved in the anti-UC effects of Iso, although direct mechanism validation is needed.

### Isorhamnetin in DSS-induced UC mice

**Fig. 9:**
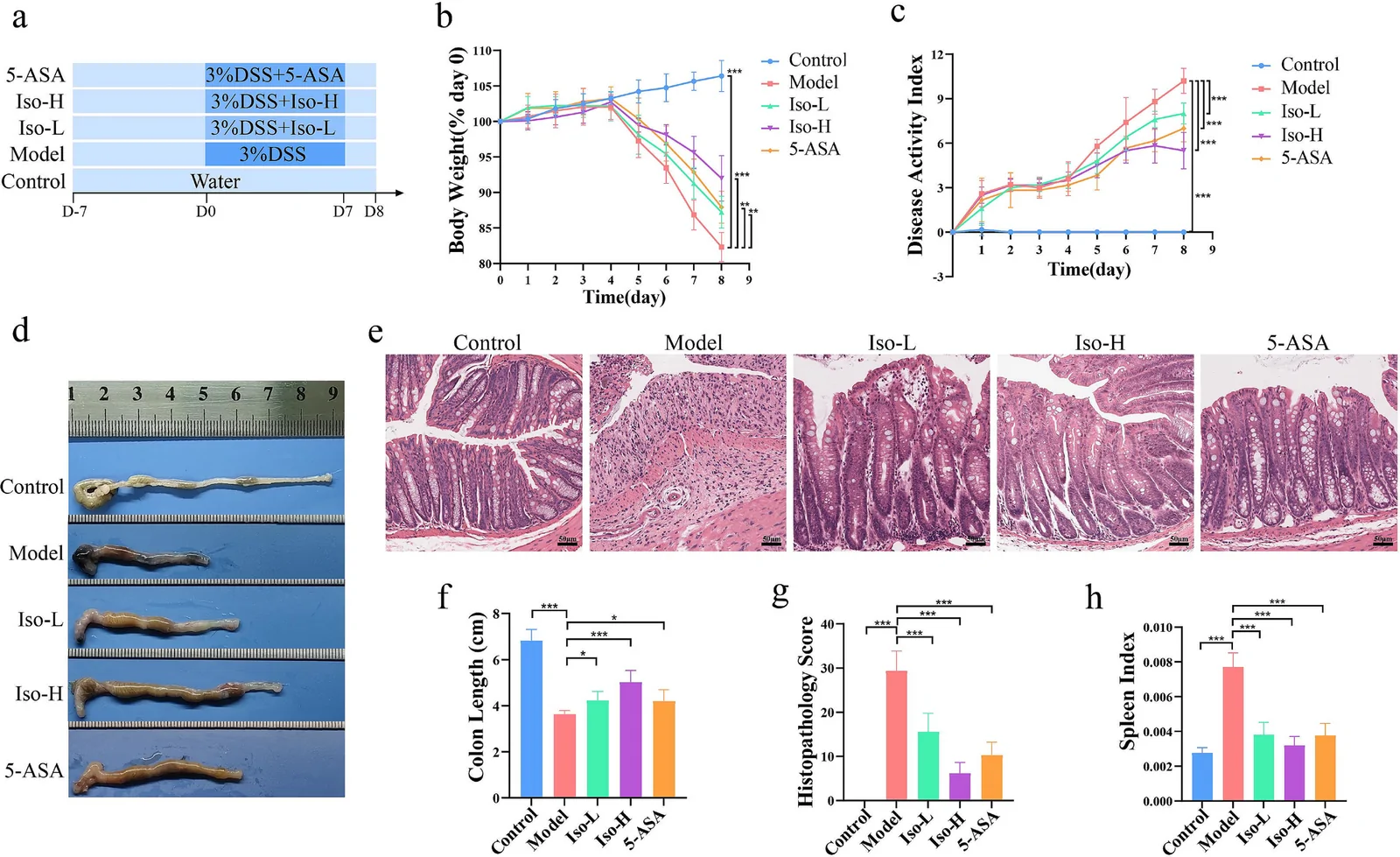
Effects of Isorhamnetin on UC. (**a**) Schematic of the mouse in vivo experiment. (**b**) Body weight changes of mice. (**c**) DAI score. (**d**, **f**) Colon length. (**e**) Representative histology of colon tissue (H&E). (**g**) Colonic histopathological score. (**h**) Splenic index. Data: mean ± SD. Group sizes: Model and Iso-L groups, *n *= 5; Control, Iso-H, and 5-ASA groups, *n* = 6. *p < 0.05, **p < 0.01, ***p < 0.001

To determine whether Iso could ameliorate UC, we examined the efficacy of Iso in vivo. Based on previous literature, two treatment groups were established: Iso-L (25 mg/kg) and Iso-H (50 mg/kg). Active UC was induced in mice by 7 days of 3% DSS, with Iso given concurrently (Fig. 9a). During the experiment, one mouse died in both the Model group and the Iso-L group, resulting in a sample size of *n* = 5 for these two groups and *n* = 6 for the other three groups. We found that, except for the Control group, all groups showed weight loss starting on Day 5. Compared to the Model group, the Iso-L and Iso-H groups had a more gradual decline in body weight, and the Iso-H group appeared to have a greater efficacy. These findings suggest that Iso may alleviates weight loss in UC mice depending on the dose (Iso-L vs. Model: MD 4.91, 95% CI 1.70, 8.11; Iso-H vs. Model: MD 9.67, 95% CI 6.60, 12.74) (Fig. 9b). The DAI score further indicated that Iso reduced the grade of diarrhea and fecal bleeding in UC mice, especially in the Iso-H group, where the DAI score showed a significant reduction relative to the Model group (Iso-L vs. Model: MD −2.20, 95% CI −3.30, −1.10; Iso-H vs. Model: MD −4.70, 95% CI −5.75, −3.65) (Fig. 9c). UC caused colon shortening, which was ameliorated by Iso. The Iso-H group showed the most pronounced restoration of colon length (Iso-L vs. Model: MD 0.60, 95% CI 0.03, 1.17; Iso-H vs. Model: MD 1.38, 95% CI 0.83, 1.92) (Fig. 9d, f). H&E staining revealed extensive mucosal damage in colons of the Model group: loss of mucosal integrity, crypt destruction, and inflammatory cell infiltration. In contrast, Iso appeared to preserve colonic mucosal integrity, improved crypt structure, and attenuated inflammatory cell infiltration (Iso-L vs. Model: MD −13.80, 95% CI −17.85, −9.75; Iso-H vs. Model: MD −23.23, 95% CI −27.11, −19.35) (Fig. 9e, g). DSS-induced colitis in mice resulted in an elevated splenic index (calculated as spleen weight relative to body weight), suggesting increased systemic inflammation (Zhou et al. 2024). However, administration of Iso significantly reduced the splenic index (Iso-L vs. Model: MD −0.0039, 95% CI −0.0047, −0.0031; Iso-H vs. Model: MD −0.0045, 95% CI −0.0053, −0.0038) (Fig. 9h). In conclusion, these results suggest that Iso may ameliorate DSS‑induced UC in a dose‑dependent manner.

## Discussion

We selected Iso from the clinical evidence through a multi-step screening strategy. First, an NMA of 17 RCTs ranked the ten TCM formulations with the highest SUCRA values for active UC. Second, association rule analysis of these top-ranked formulations revealed eight core Chinese herbs that frequently occurred in these formulations. Third, network pharmacology analysis was used to study these eight herbs. A topological analysis of the “Chinese herb – component – target” network ranked the components by their “Degree” scores, yielding quercetin, stigmasterol, kaempferol, beta-sitosterol, and Iso as the top five core active components. Fourth, molecular docking against the three key targets (IL1B, PTGS2, PPARG) showed that Iso exhibited the strongest binding affinity to PTGS2 (−9.34 kcal/mol). Additional docking showed strong binding between Iso and Nrf2 (−9.16 kcal/mol). Taken together, these results—spanning clinical efficacy ranking, herbal association mining, network topological analysis, and molecular docking—pointed to Iso as a potential candidate for in vivo validation. Then, in vivo experiments showed that Iso dose-dependently ameliorated DSS-induced UC in mice, which is consistent with the predictions of our screening process, although the molecular mechanisms need further experimental investigation. These findings may provide some evidence supporting TCM for UC and could contribute to future drug development.

We included 17 RCTs (1,598 patients) for meta-analysis, showing that TCM significantly decreased the Mayo score. Our SUCRA rankings provide a basis for further screening of core formulas, suggesting that formulas such as QHG, SP, and MWMP have a high probability of efficacy (Wang et al. 2022; Wu et al. 2022b). These findings are consistent with the treatment principles of TCM for UC (Cao et al. 2026). However, the high heterogeneity (*I*^2^ = 94%) remains, which is likely attributable to observable variability across studies in disease severity, treatment duration, concomitant therapies, and herbal formulations. Although we applied a random-effects model and conducted sensitivity analyses, this heterogeneity should be considered when interpreting the treatment rankings. Furthermore, several intervention nodes were supported by only a single RCT. SUCRA rankings should therefore be viewed as hypothesis-generating rather than conclusive, and may change as additional trials become available.We found eight core Chinese herbs from the top ten formulas ranked by SUCRA through association rule analysis. Among them, *Aucklandia lappa*, *Coptis chinensis*, *Glycyrrhiza uralensis,* and *Poria cocos* are all used in the clinical management of UC. Modern pharmacological studies show that costunolide from *Aucklandia lappa* has been shown to alleviate UC by blocking the activation of the RIPK1/3-MLKL axis (Su et al. 2024). Berberine in *Coptis chinensis* can alleviate intestinal inflammation by inhibiting the PPAR-γ/mTOR/HIF-1α signaling pathway (He et al. 2026). The Chinese herbal medicine *Glycyrrhiza uralensis* alleviates colonic inflammation by inhibiting NF-κB activation, rebalancing Th17/Treg cell homeostasis, downregulating cytokine expression of IL-1β, IL-17, and TNF-α, and upregulating IL-10 expression (Kong et al. 2024; Shi et al. 2022). *Poria cocos* polysaccharide enhances colonic barrier function by promoting tight junction protein levels. Moreover, it exerts anti-inflammatory effects by inhibiting NF-κB activation (Wan et al. 2024). However, the “multi-component, multi-target” characteristics of TCM formulas make the analysis of their mechanisms particularly complex. Therefore, we employed network pharmacology to investigate the underlying mechanism of these core Chinese herbs. We found that the 146 active components in these Chinese herbs act on 76 UC-related targets, suggesting a potential “network-based” mechanism to treat the disease. PPI analysis further selected ten core targets such as IL1B, TNF, IL-6, PTGS2, and PPARG, which are associated with regulating immune-inflammatory responses and are consistent with the pathogenesis of UC (Atreya and Neurath 2026; Jiang et al. 2025; Chen et al. 2026b; Gao et al. 2026).

To further explore key targets, we performed a Venn analysis between the core targets and DEGs in UC patients' colonic tissues. IL1B, PTGS2 and PPARG were identified as three key targets. IL1B, a key IL-1 family member, is significantly overexpressed in the UC colonic mucosa, serving as a core factor that contributes to the inflammatory cascade reaction (Lin et al. 2026). PTGS2 is a key enzyme in prostaglandin synthesis, and is highly expressed in inflamed tissues, representing a potential therapeutic target for UC (Tang et al. 2025). PPARG, a nuclear receptor, is involved in regulating adipocyte differentiation and inflammatory responses (Gu et al. 2026). It has been reported that targeting PPARG may alleviate DSS-induced UC (Xie et al. 2025).

**Fig. 10:**
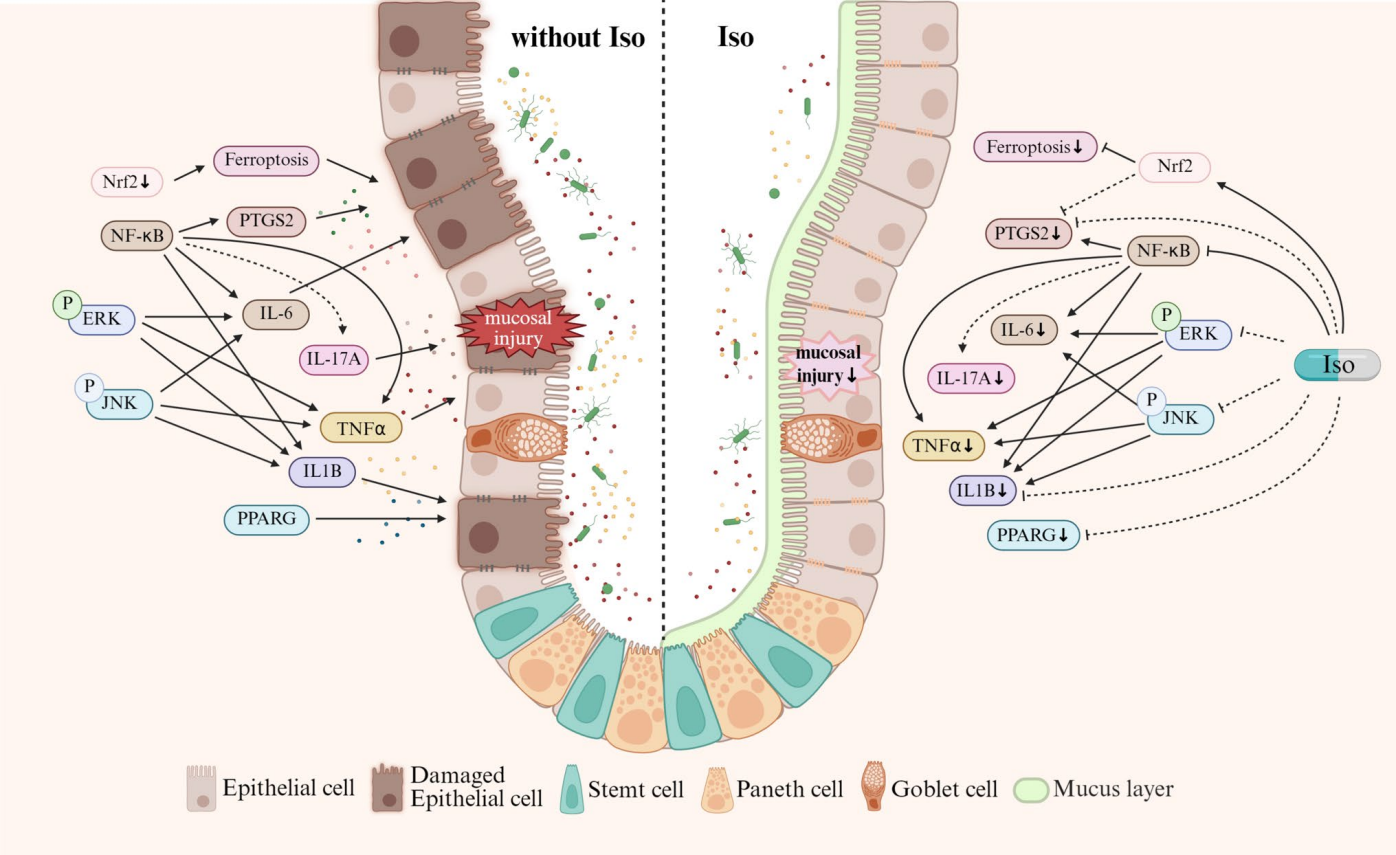
Mechanism diagram of Isorhamnetin in alleviating UC. Isorhamnetin alleviates UC, which may involve regulating the NF-κB, MAPK, and IL-17 signaling pathways and targeting Nrf2, PTGS2, IL1B, and PPARG. Solid lines represent mechanisms supported by published experimental evidence; dashed lines represent interactions predicted by this study that await experimental validation. Nrf2, Nuclear factor erythroid 2-related factor 2; PTGS2, prostaglandin-endoperoxide synthase 2; IL1B, interleukin-1 beta; PPARG, peroxisome proliferator-activated receptor gamma; TNF-α, tumor necrosis factor alpha; IL-6, interleukin-6; IL-17A, interleukin-17A; NF-κB, nuclear factor kappa B; p-ERK, phosphorylated extracellular signal-regulated kinase; p-JNK, phosphorylated c-Jun N-terminal kinase

We identified the top five active components based on degree values through topological analysis of the “Chinese herb – component – target” network, such as quercetin, kaempferol, and Iso. These flavonoids have well-known anti-inflammatory, antioxidant, and immunomodulatory activities (Gangwar et al. 2025; Chen et al. 2026a; Gai et al. 2026). Molecular docking showed that the binding energy of Iso to PTGS2 was the strongest (−9.34 kcal/mol), forming multiple stable hydrogen bonds, suggesting that Iso may be a potential inhibitor targeting PTGS2. Iso is a key active component in Chinese herbs such as *Hippophae rhamnoides* and *Ginkgo biloba* (Guo et al. 2026). In recent years, the potential value of Iso has gradually attracted attention due to its ability to regulate multiple immune and inflammatory signaling pathways (Yang et al. 2024). Studies have reported that the core mechanism of Iso's anti-inflammatory effect involves its targeting of the NF-κB, MAPK and IL-17 signaling pathways. Iso has been shown to suppress the inflammatory response by inhibiting the NF-κB and MAPK signaling pathways, and downregulating the expression of pro-inflammatory mediators such as IL1B and PTGS2 (Gong et al. 2020; Chirumbolo 2014; Dou et al. 2014). In addition, Iso shows anti-inflammatory effects by inhibiting the IL-17 and NF-κB signaling pathways and regulating the Th1/Th17 cells in the spleen (Wu et al. 2022a) (Fig. 10).

Two previous studies have reported that Iso ameliorates UC (Ru et al. 2024; Zhang et al. 2025). Both studies found that Iso alleviates UC through Nrf2 activation and ferroptosis inhibition. These findings, together with our data, support the anti-UC efficacy of Iso and point to Nrf2 as a shared mechanistic target. While previous work focused on ferroptosis, our study suggests that PTGS2 may be another downstream target of Nrf2 activation. Our data predict that Iso may activate Nrf2 to inhibit PTGS2 expression, providing a potential anti-inflammatory mechanism that may act in parallel with ferroptosis suppression (Fig. 10).

Based on the above predictions, we further evaluated the efficacy of Iso on UC through in vivo experiments. The results showed that Iso dose-dependently ameliorated body weight loss, improved colon length, diminished DAI score, and lowered spleen index in UC mice. Histopathological results showed that Iso significantly preserved colonic mucosal integrity and reduced crypt damage and inflammatory cell infiltration. In terms of the mechanism, the therapeutic effect of Iso may be associated with the Nrf2-PTGS2 axis. KEGG analysis results also predict that the IL-17, NF-κB, and MAPK signaling pathways may be the important pathways through which Iso shows effects. However, the mechanism needs further experimental investigation.

This study follows a systematic research approach that links clinical evidence to experimental validation. We began with a meta-analysis of RCTs to assess the efficacy of TCM for active UC. Then, we screened core herbs and active components through association rule analysis, network pharmacology, and molecular docking. Finally, we tested the key component in an animal model. This “clinic-to-laboratory” approach offers a replicable framework for TCM research: clinical data guide compound discovery, computational tools predict targets, and in vivo experiments test efficacy. The same approach may be applied to other herbal formulas and diseases.

However, several limitations exist. First, some RCTs included in the meta-analysis did not provide detailed information on randomization and blinding, which may lead to bias. Second, the meta-analysis had a star-shaped network structure, and most treatment comparisons were based on indirect evidence without formal assessment of consistency between direct and indirect evidence; treatment rankings should therefore be interpreted cautiously. The high heterogeneity, despite the use of a random-effects model and sensitivity analyses, further supports this cautious interpretation. Moreover, several nodes were supported by only a single RCT, and SUCRA rankings should be viewed as hypothesis-generating rather than conclusive. Third, this study observed the efficacy of Iso on active UC for 7 days, and its long-term effects require further investigation. Fourth, the Nrf2-PTGS2 axis was inferred from predictive analyses and published literature without direct experimental evidence in this study, and further validation through molecular biological experiments is needed.

## Conclusion

This study systematically explored the clinical value of TCM in treating active UC and the mechanism of its core active component by integrating clinical evidence, bioinformatics predictions, and experimental validation. Our findings suggest that the anti-UC effects of Iso may involve the Nrf2-PTGS2 axis and immune-inflammatory pathways, although direct mechanistic validation remains to be performed. The integrative approach of the study may serve as a reference for future TCM research.

## Supplementary information

Below is the link to the electronic supplementary material.

ESM 1 (DOCX 95.3 KB)

ESM 2 (DOCX 35.3 KB)

ESM 3 (DOCX 82.7 MB)

## Data Availability

All data generated or analyzed during this study are included in this published article and supplementary information files. Publicly available datasets analyzed in this study include GEO Series GSE87466 and GSE206285.
