# Supplementary material for: Isorhamnetin ameliorates active ulcerative colitis potentially involving the Nrf2-PTGS2 axis: an integrative study from clinical evidence mining to in vivo validation
Source: Naunyn Schmiedebergs Arch Pharmacol. 2026 Jul 25;399(13):21667–84. doi: 10.1007/s00210-026-05737-4 (PMC13695691; doi:10.1007/s00210-026-05737-4)
Supplement: Supplementary file 3 [file 210_2026_5737_MOESM3_ESM.docx]

**Isorhamnetin Ameliorates Active Ulcerative Colitis via the Nrf2-PTGS2** **axis: An Integrative Study from Clinical Evidence Mining to In Vivo Validation**

Zhenjia Fan, Luqing Zhao, Ning Ding, Yichong Wang, Danyan Li, Shengsheng Zhang*

*Corresponding author: Shengsheng Zhang. zhangshengsheng@bjzhongyi.com. Digestive Disease Center, Beijing Hospital of Traditional Chinese Medicine, Capital Medical University, Beijing 100010, China.

**Supporting information captions**

Supplementary Table1 Search strategy of the study

Supplementary Table2. Node definitions and herbal compositions in the network meta-analysis

Supplementary Table3 Scoring systems for disease activity index (DAI)

Supplementary Table4 Criteria of histological scores for colitis

Supplementary Table5 Characteristics of included studies

Supplementary Table6 Ranking Probability of TCM Treatment Efficacy for active UC

Supplementary Fig1 Network plots for included studies

**Supplementary Fig2 Risk of bias summary**

**Supplementary Fig3 Publication bias of the meta-analysis**

Supplementary Fig4 Results of SUCRA

**Supplementary Table1** Search strategy of the study

| **Database** | **Search syntax** |
| --- | --- |
| PubMed | (((((((ulcerative colitis[MeSH Terms]) OR (inflammatory bowel disease[MeSH Terms])) OR (IBD[Title/Abstract])) OR (inflammatory bowel disease*[Title/Abstract])) OR (UC[Title/Abstract])) OR (ulcerative coliti*[Title/Abstract]))) AND ((((((((((((((((((((Herbal Medicine[MeSH Terms]) OR (Medicine, Chinese Traditional[MeSH Terms])) OR (Drugs, Chinese Herbal[MeSH Terms])) OR (Phytotherapy[MeSH Terms])) OR (Medicine, Chinese Traditional[Title/Abstract])) OR (herb*[Title/Abstract])) OR (Traditional Chinese Medicine[Title/Abstract])) OR (Traditional Medicine[Title/Abstract])) OR (Chinese Medicine[Title/Abstract])) OR (conservative treatment[Title/Abstract])) OR (conservative care[Title/Abstract])) OR (conservative therapy[Title/Abstract])) OR (TCM[Title/Abstract])) OR (Phytotherap*[Title/Abstract])) OR (Tang[Title/Abstract])) OR (Decoction[Title/Abstract])) OR (Fang[Title/Abstract])) OR (Wan[Title/Abstract])) OR (Keli[Title/Abstract])) OR (San[Title/Abstract])))) AND (((((((Randomized Controlled Trial[Publication Type]) OR (Controlled Clinical Trial[Publication Type])) OR (Pragmatic Clinical Trial[Publication Type])) OR (Clinical Trials as Topic[MeSH Terms])) OR (trial*[Title/Abstract])) OR (random*[Title/Abstract])) OR (placebo[Title/Abstract])) |
| Embase | (ulcerative colitis'/exp OR 'inflammatory bowel disease'/exp OR ibd:ti,ab,kw OR 'inflammatory bowel disease*':ti,ab,kw OR uc:ti,ab,kw OR 'ulcerative coliti*':ti,ab,kw) AND (herbal medicine'/exp OR 'medicine, chinese traditional'/exp OR 'phytotherapy'/exp OR 'medicine, chinese traditional':ti,ab,kw OR 'traditional chinese medicine':ti,ab,kw OR herb*:ti,ab,kw OR 'traditional medicine':ti,ab,kw OR 'chinese medicine':ti,ab,kw OR 'conservative treatment':ti,ab,kw OR 'conservative care':ti,ab,kw OR 'conservative therapy':ti,ab,kw OR tcm:ti,ab,kw OR phytotherap*:ti,ab,kw OR tang:ti,ab,kw OR fang:ti,ab,kw OR decoction:ti,ab,kw OR wan:ti,ab,kw OR keli:ti,ab,kw OR san:ti,ab,kw) AND (randomized controlled trial'/exp OR (controlled:ti,ab,kw AND (study:ti,ab,kw OR design:ti,ab,kw OR trial:ti,ab,kw)) OR 'intermethod comparison':ti,ab,kw OR placebo:ti,ab,kw OR random*:ti,ab,kw) |
| Web of Science | (TS=(ulcerative colitis OR inflammatory bowel disease) OR TI=(IBD OR inflammatory bowel disease* OR UC OR ulcerative coliti*)) AND (TS=(Herbal Medicine OR Medicine, Chinese Traditional OR Drugs, Chinese Herbal OR Phytotherapy) OR TI=(Medicine, Chinese Traditional OR Traditional Chinese Medicine OR herb* OR Traditional Medicine OR Chinese Medicine OR conservative treatment OR conservative care OR conservative therapy OR TCM OR Phytotherap* OR Tang OR Decoction OR Fang OR Wan OR San) OR AB=(Medicine, Chinese Traditional OR Traditional Chinese Medicine OR herb* OR Traditional Medicine OR Chinese Medicine OR conservative treatment OR conservative care OR conservative therapy OR TCM OR Phytotherap* OR Tang OR Decoction OR Fang OR Wan OR San)) AND (TS=(Randomized Controlled Trial OR Clinical Trial) OR TI=(trial* OR ((controlled) and (study or design or trial)) OR intermethod comparison OR random* OR placebo) OR AB=(trial* OR ((controlled) and (study or design or trial)) OR intermethod comparison OR random* OR placebo)) |
| Cochrane | ((MeSH descriptor: [Colitis, Ulcerative] explode all trees) or (MeSH descriptor: [Inflammatory Bowel Diseases] explode all trees) or (IBD):ti,ab,kw or (inflammatory bowel disease*):ti,ab,kw or (UC):ti,ab,kw or (ulcerative coliti*):ti,ab,kw) and ((MeSH descriptor: [Herbal Medicine] explode all trees) or (MeSH descriptor: [Medicine, Chinese Traditional] explode all trees) or (MeSH descriptor: [Drugs, Chinese Herbal] explode all trees) or (MeSH descriptor: [Phytotherapy] explode all trees) or (Medicine, Chinese Traditional):ti,ab,kw or (Traditional Chinese Medicine):ti,ab,kw or (herb*):ti,ab,kw or (Traditional Medicine):ti,ab,kw or (Chinese Medicine):ti,ab,kw or (conservative treatment):ti,ab,kw or (conservative care):ti,ab,kw or (conservative therapy):ti,ab,kw or (TCM):ti,ab,kw or (Phytotherap*):ti,ab,kw or (Tang):ti,ab,kw or (Fang):ti,ab,kw or (Decoction):ti,ab,kw or (Wan):ti,ab,kw or (keli):ti,ab,kw or (San):ti,ab,kw) |
| CNKI | (SU=('溃疡性结肠炎'+'炎症性肠病') or TKA=('IBD'+'UC'+'溃疡性结肠炎'+'炎症性肠病'+'溃结')) and (SU=('中医'+'中药'+'中医药'+'草药'+'中草药'+'方剂'+'药方'+'颗粒'+'汤药'+'中成药')) and (SU=('随机对照试验'+'随机对照研究'+'RCT'+'随机')) |
| Wanfang | (主题：("溃疡性结肠炎" or "炎症性肠病") or 题名或关键词：("IBD" or "UC" or "溃疡性结肠炎" or "炎症性肠病" or "溃结")) and (主题：("中医" or "中药" or "中医药" or "草药" or "中草药" or "方剂" or "药方" or "颗粒" or "汤药" or "中成药")) and (主题：("随机对照试验" or "随机对照研究" or "RCT")) |
| VIP | M=(溃疡性结肠炎 OR 炎症性肠病 OR IBD OR UC OR 溃结) AND (中医 OR 中药 OR 中医药 OR 草药 OR 中草药 OR 方剂 OR 药方 OR 颗粒 OR 汤药 OR 中成药) AND (随机对照试验 OR 随机对照研究 OR RCT OR 随机) |

**Supplementary Table2**. Node definitions and herbal compositions in the network meta-analysis

| **Node Code** | **Formula Name** | **Herbal Composition** | **Number of Studies** |
| --- | --- | --- | --- |
| SBG | Shenling Baizhu Granule | Panax ginseng (Ren Shen), Poria cocos (Fu Ling), Atractylodes macrocephala (Bai Zhu), Dioscorea opposita (Shan Yao), Lablab purpureus (Bai Bian Dou), Nelumbo nucifera (Lian Zi), Coix lacryma-jobi (Yi Yi Ren), Amomum villosum (Sha Ren), Platycodon grandiflorus (Jie Geng), Glycyrrhiza uralensis (Gan Cao). | 1 |
| SP | Sishen Pill | Psoralea corylifolia (Bu Gu Zhi), Aucklandia lappa (Mu Xiang), Myristica fragrans (Rou Dou Kou), Papaver somniferum (Ying Su Ke), Terminalia chebula (He Zi), Paeonia lactiflora (Bai Shao), Zingiber officinale (Gan Jiang), Atractylodes macrocephala (Bai Zhu), Evodia rutaecarpa (Wu Zhu Yu) | 1 |
| YJHF | Yiqi Jiedu Huayu Formula | Codonopsis pilosula (Dang Shen), Atractylodes macrocephala (Bai Zhu), Paeonia veitchii (Chi Shao), Paeonia lactiflora (Bai Shao), Aucklandia lappa (Mu Xiang), Portulaca oleracea (Ma Chi Xian), Coix lacryma-jobi (Yi Yi Ren), Bletilla striata (Bai Ji), Panax notoginseng (San Qi Fen), Setaria italica (Gu Ya), Coptis chinensis (Huang Lian), Phellodendron chinense (Huang Bai). | 1 |
| JZG | Jianpi Zhixiening Granule | Codonopsis pilosula (Dang Shen), Nelumbo nucifera (Lian Zi), Lablab purpureus (Bai Bian Dou), Coptis chinensis (Huang Lian), Scutellaria baicalensis (Huang Qin), Lonicera japonica (Jin Yin Hua), Medicated Leaven (Jian Qu), Crataegus pinnatifida (Shan Zha), Plantago asiatica (Che Qian Zi), Zingiber officinale (Gan Jiang). | 1 |
| QHYF | Qingre Huashi Yuyang Formula | Pulsatilla chinensis (Bai Tou Weng), Siegesbeckia orientalis (Xi Xian Cao), Paeonia lactiflora (Bai Shao), Smilax glabra (Tu Fu Ling), Sanguisorba officinalis (Di Yu), Coptis chinensis (Huang Lian), Phellodendron chinense (Huang Bai), Fraxinus rhynchophylla (Qin Pi), Paeonia suffruticosa (Dan Pi), Aucklandia lappa (Mu Xiang), Bletilla striata (Bai Ji), Glycyrrhiza uralensis (Gan Cao). | 1 |
| JLJM | Jianpi Lishi Jiedu Mixture | Codonopsis pilosula (Dang Shen), Smilax glabra (Tu Fu Ling), Atractylodes macrocephala (Bai Shu), Forsythia suspensa (Lian Qiao), Lonicera japonica (Ren Dong Teng), Cynanchum atratum (Bai Wei). | 1 |
| QCWZ | Qingchang Wenzhong Decoction | ***Coptis chinensis*** (Huang Lian), ***Zingiber officinale*** (Gan Jiang), ***Sophora flavescens*** (Ku Shen), ***Aucklandia lappa*** (Mu Xiang), ***Panax notoginseng*** (San Qi), ***Indigo naturalis*** (Qing Dai), Sanguisorba officinalis (Di Yu), ***Glycyrrhiza uralensis*** (Gan Cao). | **1** |
| MWMP | Wumei Pill | Prunus mume (Wu Mei), Coptis chinensis (Huang Lian), Phellodendron chinense (Huang Bai), Asarum heterotropoides (Xi Xin), Zanthoxylum bungeanum (Hua Jiao), Zingiber officinale (Gan Jiang), Cinnamomum cassia (Gui Zhi), Aconitum carmichaelii (Fu Zi), Aucklandia lappa (Mu Xiang), Sophora flavescens (Ku Shen), Indigo naturalis (Qing Dai), Panax notoginseng (San Qi), Sanguisorba officinalis (Di Yu), Pulsatilla chinensis (Bai Tou Weng), Fraxinus rhynchophylla (Qin Pi), Glycyrrhiza uralensis (Gan Cao). | 1 |
| WJD | Wenglian Jiedu Decoction | Coptis chinensis (Huang Lian), Pulsatilla chinensis (Bai Tou Weng), Sophora flavescens (Ku Shen), Taraxacum mongolicum (Pu Gong Ying), Phellodendron chinense (Huang Bai), Fraxinus rhynchophylla (Qin Pi), Sanguisorba officinalis (Di Yu), Rhodiola rosea (Hong Jing Tian), Pueraria lobata (Ge Gen), Sargentodoxa cuneata (Da Xue Teng), Citri Reticulatae Pericarpium (Chen Pi), Cimicifuga heracleifolia (Sheng Ma), Saposhnikovia divaricata (Fang Feng), Paeonia lactiflora (Bai Shao), Poria cocos (Fu Ling), Atractylodes macrocephala (Bai Zhu), Coix lacryma-jobi (Yi Yi Ren), Glycyrrhiza uralensis (Gan Cao). | 1 |
| IN | Indigo Naturalis | Indigo naturalis (Qing Dai). | 1 |
| A. paniculata | Andrographis paniculata | Andrographis paniculata (Chuan Xin Lian). | 1 |
| GXD | Gancao Xiexin Decoction | Glycyrrhiza uralensis (Gan Cao), Codonopsis pilosula (Dang Shen), Ziziphus jujuba (Da Zao), Scutellaria baicalensis (Huang Qin), Pinellia ternata (Ban Xia), Zingiber officinale (Gan Jiang), Coptis chinensis (Huang Lian). | 1 |
| SPC | SishenPill Compound | Atractylodes macrocephala (Bai Zhu), Myristica fragrans (Rou Dou Kou), Psoralea corylifolia (Bu Gu Zhi), Schisandra chinensis (Wu Wei Zi), Zingiber officinale (Gan Jiang), Cimicifuga heracleifolia (Sheng Ma), Platycodon grandiflorus (Jie Geng), Bupleurum chinense (Chai Hu), Poria cocos (Fu Ling), Dioscorea opposita (Shan Yao), Pulsatilla chinensis (Bai Tou Weng), Smilax glabra (Tu Fu Ling). | 1 |
| BZD | Buyang Zhixie Decoction | Psoralea corylifolia (Bu Gu Zhi), Myristica fragrans (Rou Dou Kou), Coptis chinensis (Huang Lian), Corchorus capsularis (Huang Ma Gen), Dioscorea opposita (Shan Yao), Artemisia anomala (Liu Ji Nu), Aucklandia lappa (Mu Xiang), Acorus calamus (Chang Pu), Massa Medicata Fermentata (Shen Qu), Hordeum vulgare (Mai Ya), Crataegus pinnatifida (Shan Zha), Glycyrrhiza uralensis (Gan Cao). | 1 |
| HSD | Huangqin Shaoyao Decoction | Astragalus membranaceus (Huang Qi), Scutellaria baicalensis (Huang Qin), Codonopsis pilosula (Dang Shen), Paeonia lactiflora (Shao Yao), Lonicera japonica (Jin Yin Hua), Ziziphus jujuba (Da Zao), Glycyrrhiza uralensis (Gan Cao), Atractylodes macrocephala (Bai Zhu), Angelica sinensis (Dang Gui), Coptis chinensis (Huang Lian), Aucklandia lappa (Mu Xiang). | 1 |
| QJD | QingreJianpi Decoction | Patrinia scabiosifolia (Bai Jiang Cao), Coix lacryma-jobi (Yi Yi Ren), Portulaca oleracea (Ma Chi Xian), Halloysitum Rubrum (Chi Shi Zhi), Poria cocos (Fu Ling), Scutellaria baicalensis (Huang Qin), Pueraria lobata (Ge Gen), Codonopsis pilosula (Dang Shen), Atractylodes macrocephala (Bai Zhu), Paeonia lactiflora (Bai Shao), Aucklandia lappa (Mu Xiang), Citri Reticulatae Pericarpium (Chen Pi), Coptis chinensis (Huang Lian), Glycyrrhiza uralensis (Gan Cao). | 1 |
| QHG | Qingre Huashi Granule | Taraxacum mongolicum (Pu Gong Ying), Pulsatilla chinensis (Bai Tou Weng), Sanguisorba officinalis (Di Yu), Areca catechu (Bing Lang), Sophora japonica (Huai Hua), Bletilla striata (Bai Ji), Paeonia veitchii (Chi Shao). | 1 |

**Supplementary Table3** Scoring systems for disease activity index (DAI)

| **Score** | **Weight loss** | **Stool consistency** | **Rectal bleeding** |
| --- | --- | --- | --- |
| 0 | None | Normal | Negative hemoccult |
| 1 | 1-5% | Soft but still formed |  |
| 2 | 6-10% | Soft | Positive hemoccult |
| 3 | 11-18% | Very soft, wet | Blood trace in stool visible |
| 4 | > 18% | Watery diarrhea | Gross rectal bleeding |

**Supplementary Table4** Criteria of histological scores for colitis

| **Score** | **Severity of inflammation** | **Extent of injury** | **Crypt damage** |
| --- | --- | --- | --- |
| 0 | None | None | None |
| 1 | Mild | Mucosal | Basal 1/3 damaged |
| 2 | Moderate | Mucosal and submucosal | Basal 2/3 damaged |
| 3 | Severe | Transmural | Only have intact epithelial surface |
| 4 | - | - | Entire crypt and epithelium loss |

Each score was multiplied by the coefficient based on percentage of tissue involved: 0–25%:

×1; 26–50%: ×2; 51–75%: ×3; 76–100%: ×4. The range of histological score was 0-40.

**Supplementary Table5** Characteristics of included studies

| **No.** | **Study** | **Intervention** | **Number of patients** | **Sex male/**  **female** | **Ages (**$\bar{\mathbf{x}}$**± s)** | **Course of disease**  **(years)** | **Duration (days)** | **Severity grading** |
| --- | --- | --- | --- | --- | --- | --- | --- | --- |
| 1 | Bai 2025 | SBG | 51 | 21/30 | 52.31±5.46 | 3.22±0.83 | 90 | 16 MiUCs, 35 MoUCs |
|  |  | C: 5-ASA | 51 | 19/32 | 52.01±5.14 | 3.04±0.79 | 90 | 18 MiUCs, 33 MoUCs |
| 2 | Cheng 2018 | SP | 92 | 48/44 | 47.5±16.8 | 3.5±0.8 | 84 | -  - |
|  |  | C: 5-ASA | 92 | 50/42 | 46.3±16.5 | 3.6±0.7 | 84 |  |
| 3 | ChenJL 2021 | YJHF | 30 | 16/14 | 37.8±3.2 | 1.3±0.2 | 60 | 18 MiUCs, 12 MoUCs |
|  |  | C: 5-ASA | 30 | 15/15 | 34.1±3.4 | 1.4±0.3 | 60 | 20 MiUCs, 10 MoUCs |
| 4 | ChenL 2021 | JZG | 58 | 34/24 | 59.32±6.75 | 6.93±2.04 | 60 | 25 MiUCs, 33 MoUCs |
|  |  | C: 5-ASA | 57 | 32/25 | 59.01±6.82 | 6.74±2.25 | 60 | 27 MiUCs, 30 MoUCs |
| 5 | Hu 2024 | QHYF | 75 | 39/36 | 35.9±5.7 | 6.5±1.5 | 56 | 46 MiUCs, 23 MoUCs, 6 SUCs |
|  |  | C: 5-ASA + Corticosteroids | 75 | 41/34 | 36.3±5.9 | 5.9±1.7 | 56 | 48 MiUCs, 22 MoUCs, 5 SUCs |
| 6 | Huang 2025 | JLJM | 36 | 18/20 | 48.9±10.1 | 3.46±1.40 | 90 | 17 MiUCs, 21 MoUCs |
|  |  | C: 5-ASA | 36 | 21/18 | 45.3±12.3 | 3.22±1.42 | 90 | 16 MiUCs, 23 MoUCs |
| 7 | Li 2025-1 | QCWZ | 29 | 22/7 | 38.6 | 3.4 | 56 | MoUCs |
|  |  | C: 5-ASA | 32 | 21/11 | 39.7 | 4.9 | 56 |  |
| 8 | Li 2025-2 | MWMP | 8 | 2/6 | 42.6 | 5.3 | 168 | SUCs |
|  |  | C: 5-ASA + Corticosteroids | 7 | 5/2 | 45.8 | 3.4 | 168 |  |
| 9 | Liu 2020 | WJD | 30 | 21/9 | 29±6 | - | 90 | 23 MiUCs, 36 MoUCs, 1 SUCs |
|  |  | C: 5-ASA | 30 | 20/10 | 27±5 | - | 90 |  |
| 10 | Naganuma 2018 | IN | 21 | 9/12 | 35.3±14.8 | 4 | 56 | MoUCs, SUCs |
|  |  | C: NO | 22 | 11/11 | 40.8±12.8 | 7 | 56 |  |
| 11 | Sandborn 2013 | A. paniculata | 74 | 41/33 | 45.6±13.6 | 6.1±6.2 | 56 | MiUCs, MoUCs |
|  |  | C: 5-ASA/NO | 75 | 41/34 | 44.7±15.2 | 5.3±5.9 | 56 |  |
| 12 | Sun 2018 | GXD | 47 | 21/26 | 46.41±5.63 | 2.65±0.87 | 42 | 15 MiUCs, 24 MoUCs, 8 SUCs |
|  |  | C: 5-ASA | 47 | 22/25 | 47.32±5.46 | 2.73±0.92 | 42 | 17 MiUCs, 23 MoUCs, 7 SUCs |
| 13 | Wan 2023 | SPC | 68 | 37/31 | 43.15±16.34 | 2.51±2.31 | 84 | 40 MiUCs, 28 MoUCs |
|  |  | C: 5-ASA | 68 | 35/33 | 42.73±17.21 | 2.24±2.43 | 84 | 39 MiUCs, 29 MoUCs |
| 14 | Xi 2021 | BZD | 35 | 33/13 | 41.62±10.9 | 3.25±1.99 | 28 | 16 MiUCs, 19 MoUCs |
|  |  | C: 5-ASA | 35 | 25/10 | 40.97±10.7 | 3.27±1.92 | 28 | 15 MiUCs, 20 MoUCs |
| 15 | Yang 2025 | HSD | 49 | 29/20 | 42.37±7.52 | 5.71±1.86 | 84 | -  - |
|  |  | C: 5-ASA | 49 | 27/22 | 40.78±8.14 | 5.58±1.76 | 84 |  |
| 16 | Zhao 2021 | QJD | 42 | 29/13 | 41.15±7.58 | 4.12±1.14 | 28 | 18 MiUCs, 24 MoUCs |
|  |  | C: 5-ASA | 42 | 27/15 | 41.22±7.61 | 4.35±1.20 | 28 | 15 MiUCs, 27 MoUCs |
| 17 | Zhou 2024 | QHG | 60 | 31/29 | 54.36±18.37 | - | 30 | MiUCs, MoUCs |
|  |  | C: 5-ASA | 60 | 34/26 | 48.52±14.83 | - | 30 |  |

5-ASA: 5-aminosalicylic acid preparations; A. paniculata: Andrographis paniculata; BZD: Buyang Zhixie Decoction; C: conventional treatment; GXD: Gancao Xiexin Decoction; HSD: Huangqin Shaoyao Decoction; IN: Indigo Naturalis; JLJM: Jianpi Lishi Jiedu Mixture; JZG: Jianpi Zhixiening Granule; MWMP: Wumei Pill; QCWZ: Qingchang Wenzhong Decoction; QHG: Qingre Huashi Granule; QHYF: Qingre Huashi Yuyang Formula; QJD: QingreJianpi Decoction; SBG: Shenling Baizhu Granule; SP: Sishen Pill; SPC: SishenPill Compound; WJD: Wenglian Jiedu Decoction; YJHF: Yiqi Jiedu Huayu Formula.; MiUCs: Mild ulcerative colitis with Mayo score of 3-5; MoUCs: Moderate ulcerative colitis with Mayo score of 6-10; SUCs: Severe ulcerative colitis with Mayo score of 11-12.

**Supplementary Table6** Ranking Probability of TCM Treatment Efficacy for active UC

| **Rank** | **Con**  **trol** | **SBG** | **SP** | **YJ**  **HF** | **JZG** | **QH**  **YF** | **JL**  **JM** | **QCWZ** | **MWMP** | **WJD** | **IN** | **A. pani**  **culata** | **GXD** | **SPC** | **BZD** | **HSD** | **QJD** | **QHG** |
| --- | --- | --- | --- | --- | --- | --- | --- | --- | --- | --- | --- | --- | --- | --- | --- | --- | --- | --- |
| **Best** | 0 | 0 | 45 | 0 | 0 | 0 | 0 | 0 | 2.6 | 34.2 | 0 | 0 | 0 | 0 | 0 | 0 | 18.1 | 0 |
| **2nd** | 0 | 0 | 37.6 | 0 | 0 | 0 | 0 | 0 | 2.9 | 29 | 0 | 0 | 0 | 0 | 0 | 0 | 30.4 | 0.1 |
| **3rd** | 0 | 0.6 | 16.2 | 0.2 | 0 | 0.1 | 0 | 0 | 7.1 | 31 | 0 | 0 | 0 | 0 | 0 | 0 | 43.4 | 1.4 |
| **4th** | 0 | 16.2 | 1.1 | 2.9 | 0 | 5.8 | 0 | 0.2 | 25 | 4.9 | 0.2 | 0 | 0 | 0 | 0 | 0 | 7.3 | 36.2 |
| **5th** | 0 | 27.8 | 0 | 6.8 | 0 | 16.6 | 0 | 0.9 | 11.9 | 0.7 | 0.7 | 0 | 0 | 0.3 | 0.2 | 0 | 0.7 | 33.5 |
| **6th** | 0 | 26.2 | 0 | 12.3 | 0 | 28.4 | 0 | 2.9 | 9.5 | 0.1 | 1.4 | 0 | 0 | 1.6 | 0.8 | 0 | 0.1 | 16.7 |
| **7th** | 0 | 17.6 | 0 | 18.7 | 0 | 28 | 0.2 | 6.2 | 9.5 | 0 | 3 | 0 | 0 | 5.6 | 2.7 | 0 | 0 | 8.5 |
| **8th** | 0 | 7.7 | 0 | 23.1 | 0.4 | 15.4 | 0.7 | 13.5 | 8.1 | 0 | 5.9 | 0 | 0 | 16.2 | 6.2 | 0 | 0 | 2.8 |
| **9th** | 0 | 2.9 | 0 | 15.2 | 1.6 | 4.4 | 2.4 | 19.8 | 5.5 | 0 | 8.7 | 0 | 0 | 26.8 | 11.9 | 0 | 0 | 0.8 |
| **10th** | 0 | 0.6 | 0 | 9.7 | 4.1 | 0.9 | 4.6 | 20.1 | 4.9 | 0 | 12.3 | 0 | 0 | 25.1 | 17.2 | 0.1 | 0 | 0.1 |
| **11th** | 0 | 0.3 | 0 | 6 | 10.7 | 0.3 | 9.6 | 16.3 | 4.4 | 0 | 15.1 | 0 | 0.1 | 15 | 21.7 | 0.6 | 0 | 0 |
| **12th** | 0 | 0 | 0 | 2.9 | 21.4 | 0.1 | 16.9 | 10.6 | 2.8 | 0 | 16.2 | 0 | 0.7 | 6.6 | 19.3 | 2.4 | 0 | 0 |
| **13th** | 0 | 0 | 0 | 1.6 | 30.1 | 0 | 25 | 5.5 | 1.8 | 0 | 14.3 | 0 | 1.9 | 2.2 | 11.4 | 6.2 | 0 | 0 |
| **14th** | 0 | 0 | 0 | 0.5 | 23.6 | 0 | 26.4 | 3 | 2 | 0 | 13.1 | 0 | 7 | 0.6 | 6.4 | 17.5 | 0 | 0 |
| **15th** | 0 | 0 | 0 | 0.1 | 6.7 | 0 | 10.5 | 0.6 | 1 | 0 | 6.5 | 0 | 27.2 | 0.1 | 1.8 | 45.5 | 0 | 0 |
| **16th** | 0.4 | 0 | 0 | 0 | 1.3 | 0 | 3.6 | 0.3 | 0.9 | 0 | 2.7 | 0.5 | 62.3 | 0 | 0.3 | 27.7 | 0 | 0 |
| **17th** | 68.2 | 0 | 0 | 0 | 0 | 0 | 0 | 0 | 0 | 0 | 0 | 31.1 | 0.6 | 0 | 0 | 0.1 | 0 | 0 |
| **Worst** | 31.4 | 0 | 0 | 0 | 0 | 0 | 0 | 0 | 0 | 0 | 0 | 68.4 | 0.2 | 0 | 0 | 0 | 0 | 0 |

**Supplementary Fig1** Network plots for included studies. A. paniculata: Andrographis paniculata; BZD: Buyang Zhixie Decoction; C: conventional treatment; GXD: Gancao Xiexin Decoction; HSD: Huangqin Shaoyao Decoction; IN: Indigo Naturalis; JLJM: Jianpi Lishi Jiedu Mixture; JZG: Jianpi Zhixiening Granule; MWMP: Wumei Pill; QCWZ: Qingchang Wenzhong Decoction; QHG: Qingre Huashi Granule; QHYF: Qingre Huashi Yuyang Formula; QJD: QingreJianpi Decoction; SBG: Shenling Baizhu Granule; SP: Sishen Pill; SPC: SishenPill Compound; WJD: Wenglian Jiedu Decoction; YJHF: Yiqi Jiedu Huayu Formula.

**Supplementary Fig2** Risk of bias summary

**Supplementary Fig3 Publication bias of the meta-analysis**

**Supplementary Fig4** Results of SUCRA. A. paniculata: Andrographis paniculata; BZD: Buyang Zhixie Decoction; C: conventional treatment; GXD: Gancao Xiexin Decoction; HSD: Huangqin Shaoyao Decoction; IN: Indigo Naturalis; JLJM: Jianpi Lishi Jiedu Mixture; JZG: Jianpi Zhixiening Granule; MWMP: Wumei Pill; QCWZ: Qingchang Wenzhong Decoction; QHG: Qingre Huashi Granule; QHYF: Qingre Huashi Yuyang Formula; QJD: QingreJianpi Decoction; SBG: Shenling Baizhu Granule; SP: Sishen Pill; SPC: SishenPill Compound; WJD: Wenglian Jiedu Decoction; YJHF: Yiqi Jiedu Huayu Formula.
